# Anti-inflammatory medications for the treatment of mental disorders: A scoping review^☆^

**DOI:** 10.1016/j.bbih.2022.100518

**Published:** 2022-09-19

**Authors:** Rebecca Fitton, Jennifer Sweetman, William Heseltine-Carp, Christina van der Feltz-Cornelis

**Affiliations:** a Kings College London, London, United Kingdom; b Tees Esk and Wear Valley NHS Foundation Trust, Darlington, United Kingdom; c Leeds and York Partnership NHS Foundation Trust, Leeds, United Kingdom; d Dept of Health Sciences, University of York, York, United Kingdom; e Hull York Medical School (HYMS), University of York, York, United Kingdom; f Hull University Teaching Hospitals NHS Trust, United Kingdom; g Institute of Health Informatics, University College London, London, United Kingdom

**Keywords:** Inflammation, Anti-inflammatory treatment, Mental health, Somatic symptoms, Scoping review

## Abstract

This scoping review assessed the effect of anti-inflammatory medications in mental disorders. A search in Medline and the Cochrane database focusing on randomised controlled trials and systematic reviews identified 53 primary research articles, conducted in major depression, bipolar disorder, schizophrenia and somatic symptom disorders and related disorders (SSRD).

The findings suggest that there is scope to consider the use of anti-inflammatory agents in mental disorders, however, not as a one-size-fits-all solution. Treatment could be especially helpful in subgroups with evidence of baseline inflammation. Anti-inflammatory medications that seem mostly effective in bipolar disorder or major depressive disorder, such as Celecoxib, Pioglitazone and statins, may differ from the ones with indications of effectiveness in schizophrenia, such as Minocycline and Aspirin. This might suggest a different underlying mechanism for treatment success in those two main illness groups. Further studies with larger sample sizes are needed that take levels of inflammation markers into account.

## Highlights

• Review of anti-inflammatory medications in mental and somatic symptom disorders.
• Evidence of anti-inflammatory use in bipolar disorder, depression and schizophrenia.
• Different mechanisms may influence treatment success in mental disorders.

## 1 Background

There is a growing body of evidence to support the role of low-grade inflammation in the pathogenesis of mental disorders. The so called “sickness behaviour” induced by pro-inflammatory cytokines such as interleukin-1 (IL-1), interleukin-6 (IL-6) and tumour necrosis factor alpha (TNFα) includes symptoms such as malaise, fatigue, anorexia, cognitive problems and pain (Dantzer, 2009), which, if sustained, align with symptoms of mental disorders. This includes somatic symptom disorders and related disorders (SSRD) that concern physical symptoms that give rise to significant emotional, cognitive and behavioural distress to such an extent that daily functioning is impaired.

Peripheral markers of inflammation such as IL-6, TNFα and CRP (Strawbridge et al., 2015; Howren et al., 2009; Haapakoski et al., 2015; Goldsmith et al., 2016) are found to be elevated in depressed patients versus healthy controls. IL-6 decreased with antidepressant treatment whilst treatment failure was associated with persistently high TNFα (Strawbridge et al., 2015). Cytokine inhibitors used in the treatment of chronic inflammatory disorders improved depressive symptoms as a secondary outcome measure, irrespective of the improvement in the primary physical illness (Kappelmann et al., 2018).Elevated levels of proinflammatory cytokines such as IL-6, IL-8, TNFα and INF-ɣ (Miller et al., 2011; Frydecka et al., 2018; Mondelli et al., 2015) and reduced levels of anti-inflammatory cytokines such as IL-10 (Frydecka et al., 2018) have also been demonstrated in psychotic disorders and correlated with symptom severity as well as a poor response to antipsychotics (Mondelli et al., 2015; Enache et al., 2021; Kose et al., 2021).

Systemic Low-grade Inflammation(SLI) might have a potential role in functional neurological disorder (FND) and other SSRDs, given the significant association these conditions have with early life trauma and stressful life events, as well as the overlap with other comorbid mental disorders (O'Connell et al., 2020). Indeed, elevated levels of high sensitivity CRP and IL-6 were found in patients with SSRD, which was associated with increasing somatic symptoms and pain scores (van der Feltz-Cornelis et al., 2020). Elevated levels of cytokines including IL-6, IL12, IL17 and TNFα as well as microRNAs involved in inflammation, but significantly lower VEGFa and normal IL1b were found in patients with functional neurological disorder (FND) (van der Feltz-Cornelis et al., 2021). Another study of heterogeneous medically unexplained symptoms found a significant elevation in natural killer cells and B lymphocyte levels which are associated with inflammation (Houtveen et al., 2007).

These findings suggest that anti-inflammatory treatment might be helpful in treatment of mental disorders and indeed there are now a growing number of trials examining their effect. A review conducted in 2014 (Fond et al., 2014) considered the use of anti-inflammatory treatments as add-on treatments across mental disorders. Given the number of studies which have been published in this area since then, it was considered important to undertake this review and to collate the evidence for anti-inflammatory medication of all classes across all mental disorders to inform future research.

### 1.1 Aim

The aim of this review was to evaluate the effect of anti-inflammatory medications in the treatment of mental disorders including somatic symptom related disorders (SSRDs).

## 2 Methods

A scoping review (Munn et al., 2018) was conducted to identify evidence in the research literature of anti-inflammatory medications being used for anti-inflammatory purposes to treat people with mental disorders (Peters et al., 2015, 2020).

### 2.1 Search strategy

Searches were conducted in Medline and Cochrane databases up to July 2022. A complete list of search terms is included in Appendix 1. As hand searching, systematic reviews were used to identify primary research studies from their references that were not included in the randomised controlled trials search and additional relevant papers were found separately. Searches were limited to studies involving humans; no date or language limits were applied.

### 2.2 Study selection

This review focused on randomised controlled trials (RCTs) and systematic reviews (SRs) published between 1946 and July 15, 2022 in Medline, and between 1995 and July 15, 2022 in Cochrane databases.

Search results were uploaded to Rayyan software (Ouzzani et al., 2016). Duplicate studies were identified and removed. Titles and abstracts were screened against the pre-defined inclusion and exclusion criteria presented in Table 1 by three reviewers (JS, WHC and RF).

**Table 1:** Criteria for inclusion and exclusion.

Inclusion criteria	Exclusion criteria
Randomised controlled trials	Study protocols
Systematic reviews	All other study design types
Anti-inflammatory medications used for treatment	Medications associated with the development of mental disorders
Mental disorders or medically not yet explained physical health symptoms/SSRD	Alternative treatments
Adults (18 years and older)	Dietary supplements
Studies with a primary outcome assessing clinical improvement in mental health or medically not yet explained symptoms	Studies of participants under the age of 18 years

This process was piloted with dual screening completed for the initial 10% to establish reliability before reviewers completed independent screening of the remaining titles and abstracts. Screening agreement of 91–92.7% was achieved (Cohen's Kappa ranged between 0.53 and0.75) (McHugh, 2012) for the first 10% of references. After that, titles, abstracts and full text were divided and screened by JS, RF and WHC and discrepancies and uncertainties were resolved through discussion or consultation with a fourth reviewer (CFC).

### 2.3 Data synthesis

We present the results in the Tables by psychiatric condition and grouped by medication type. In the discussion, we discuss the findings and explore their clinical and research relevance.

## 3 Results

**Fig. 1:**
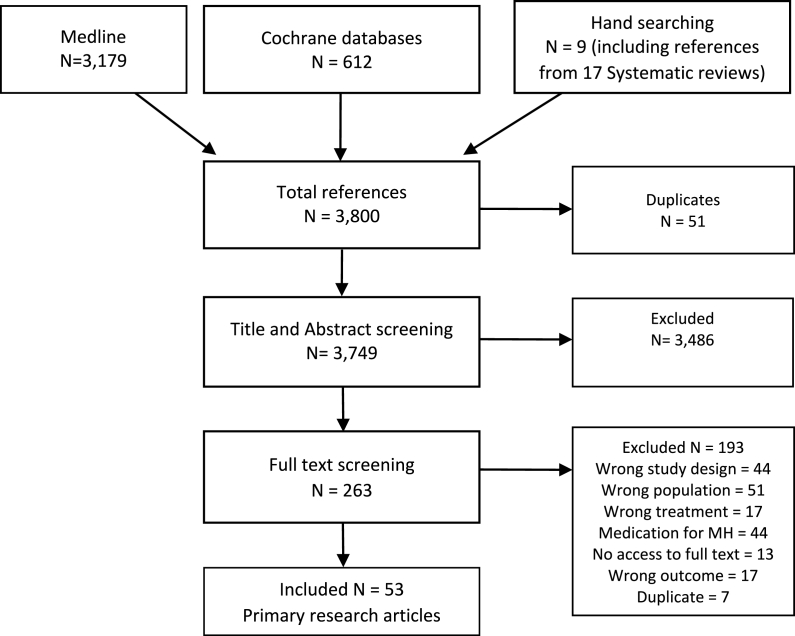
Prisma diagram.

Initial searches identified 3800 references for this review. Fig. 1 presents the full Prisma diagram showing references considered during the study selection process. Reviewers were not able to obtain full texts for 13 of the selected studies, that hence were excluded.

53 original articles reporting randomised controlled trials published between January 2002 and July 2022 were included in this review. 17 systematic reviews published between 2013 and 2021 were identified during searches (see Appendix 2). Six relevant primary research articles identified from systematic reviews were included in this review; these are shown as papers found through hand searching in Fig. 1.

The majority of references focused on depressive disorders and psychotic disorders; one considered SSRD (chronic fatigue syndrome). A range of medications were reported including non-steroidal anti-inflammatory drugs (Acetylsalicylic acid or Aspirin; Celecoxib), Minocycline, cytokine inhibitors (Anakinra; Infliximab; Tocilizumab; Adalimumab), Other (Atorvastatin; Hydroxychloroquine; Methotrexate Pentoxifylline; Pioglitazone; Pravastatin; Prednisolone; Simvastatin). Sample sizes reported for primary studies ranged between 30 and 266 participants and included studies using a range of primary outcome measures (see Appendix 3). All studies were placebo-controlled. Data from included primary research studies are presented by condition and then by type of medication.

### 3.1 Mood disorders: bipolar affective disorder

#### 3.1.1 Non-steroidal anti-inflammatory drugs

NSAIDS are competitive inhibitors of the cyclooxygenase enzyme, which converts arachidonic acid to thromboxane, prostaglandins and prostacyclin. Consequently, these molecules induce a state of inflammation via inducing hyperalgesia, hypercoagulation, fever and vasodilation (Vane and Botting, 1998). COX1 can be found predominantly within the gastric mucosa, whilst COX2 resides mostly within sites of inflammation. This makes COX2 inhibitors such as Celecoxib less prone to induce gastric bleeding which is an advantage compared to Aspirin.

#### 3.1.2 Celecoxib

As can be seen in Table 2, there were three studies assessing the efficacy of adjunctive Celecoxib medication in treatment resistant bipolar depression. Two studies evaluated Celecoxib augmentation on Escitalopram (Halaris et al., 2020; Edberg et al., 2018), the third one explored augmentation on a stable dose of a mood stabiliser or atypical antipsychotic medication (Nery et al., 2008). All three showed a greater improvement in HAM-D scores in the Celecoxib augmentation group (Halaris et al., 2020; Nery et al., 2008), although the study combining Celecoxib with a mood stabiliser or atypical antipsychotic medication only demonstrated this at one time point (week 1) and the benefit was not maintained by the study end point (Nery et al., 2008). One study assessed the efficacy of Celecoxib (400 mg daily) as an adjunctive treatment to Sodium Valproate in bipolar mania and showed a significant reduction in manic symptoms with Celecoxib compared to placebo (Arabzadeh et al., 2015).

**Table 2:**
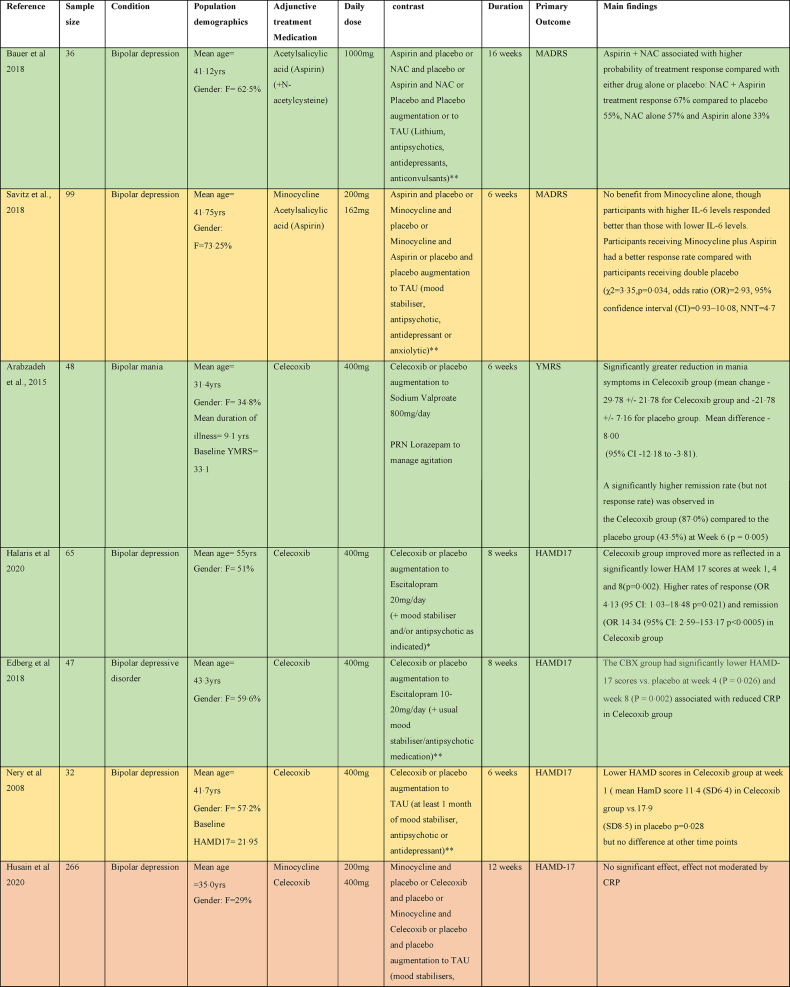

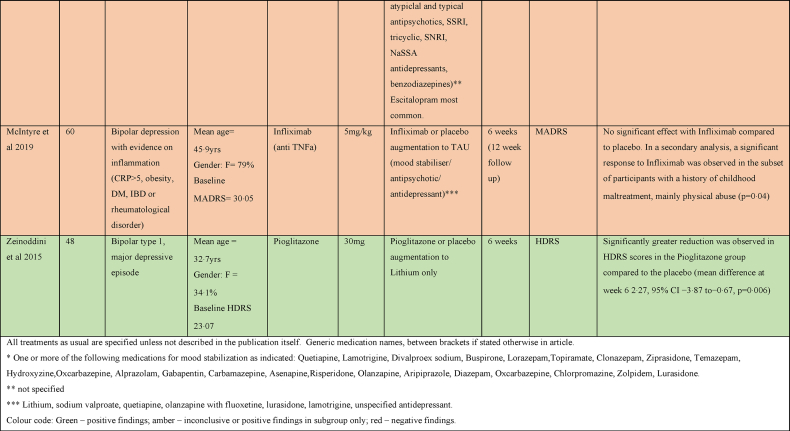
Summary of papers assessing anti-inflammatory medications in the treatment of Bipolar affective disorder (alphabetically by medication).

#### 3.1.3 Acetylsalicylic acid (Aspirin)

There was one study which assessed efficacy of Aspirin in bipolar depression, assessing it both alone and in combination with N-Acetylcysteine as an adjunct to treatment as usual (TAU) that consisted of Lithium, antipsychotics, antidepressants, or anticonvulsants. There was a modest benefit in treatment response rate in combination, but Aspirin alone did not show any benefit (Bauer et al., 2018).

#### 3.1.4 Minocycline

Within the CNS, the tetracycline antibiotic Minocycline has been shown to have anti-inflammatory, antioxidant and neuroprotective affects. In the context of inflammation of unknown origin, the mechanism remains largely unknown; however, Minocycline is thought to inhibit neutrophil migration, degranulation, oxygen-free radical production and nitric oxide release. Consequently, this inhibits glutamate mediated ecotoxicity within microglia, preventing the release of inflammatory cytokines such as IL-6 and TNFα (Elewa et al., 2006).

Two studies examined the efficacy of Minocycline in bipolar depression. Both studies examined Minocycline alone and alongside another anti-inflammatory agent (with Celecoxib or with Aspirin) as an adjunct to TAU (Husain et al., 2020; Savitz et al., 2018) that consisted of mood stabiliser, antipsychotic, antidepressant or anxiolytics. Neither study found a benefit for Minocycline alone; however, Savitz et al. found that participants receiving both Minocycline and Aspirin had a better treatment response rate than those receiving double placebo and participants with higher IL-6 levels responded better than those with lower IL-6 levels (Savitz et al., 2018).

#### 3.1.5 Cytokine inhibitors

Cytokine inhibitors refer to any agent that inhibits the function of inflammatory cytokines, thus inhibiting the inflammatory cascade, either by targeting the cytokine molecule or its receptor (Weckmann and Alcocer-Varela, 1996). This review includes anti-TNFa molecules infliximab and adalimumab, the anti interleukin-6 antibody Tocilizumab and the anti interleukin-1 molecule anakinra.

The effect of Infliximab as an adjunct to TAU with a variety of medications was explored in a study performed in 60 patients with bipolar depression and evidence of inflammation (CRP>5, obesity, DM, IBD or rheumatological disorder). No difference between Infliximab and placebo was seen overall, but a secondary analysis demonstrated a significant response to Infliximab in a subset of participants with a history of childhood maltreatment, mainly physical abuse (McIntyre et al., 2019).

#### 3.1.6 Other

Pioglitazone is prescribed in diabetes to improve the control of glucose and lipid metabolism. It has also demonstrated anti-inflammatory properties by inhibition of NF-κB (Kaplan et al., 2014). NF-κB is a protein complex involved in transcriptional induction of inflammatory chemokines, cytokines and leukocyte recruitment (Liu et al., 2017).

One study examined the efficacy of Pioglitazone in bipolar depression (in bipolar type 1 disorder) as an adjunct to Lithium (Zeinoddini et al., 2015). This found a small but significantly greater reduction in HDRS scores in the Pioglitazone group compared to placebo.

### 3.2 Mood disorders: major depressive disorder

#### 3.2.1 Celecoxib

In major depressive disorder, five studies assessed the efficacy of Celecoxib as an adjunctive treatment to antidepressant therapy (Sertraline, Reboxetine, Fluoxetine or Vortioxetine) as shown in Table 3. Four studies showed a greater decrease in HAMD score with Celecoxib (Abbasi et al., 2012; Akhondzadeh et al., 2009; Muller et al., 2006; Majd et al., 2015), although one study only demonstrated this at week 4 and by the study end point at week 8 the difference from placebo was no longer statistically significant (Majd et al., 2015). One study found no significant difference between treatment groups overall (Baune et al., 2021).

**Table 3:**
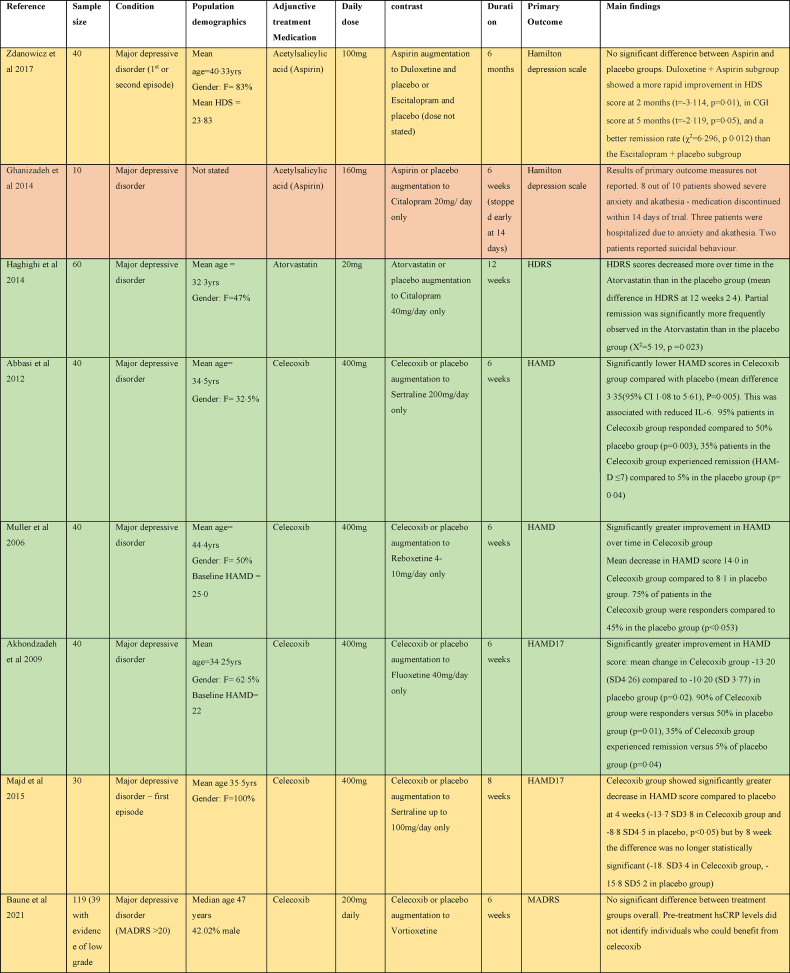

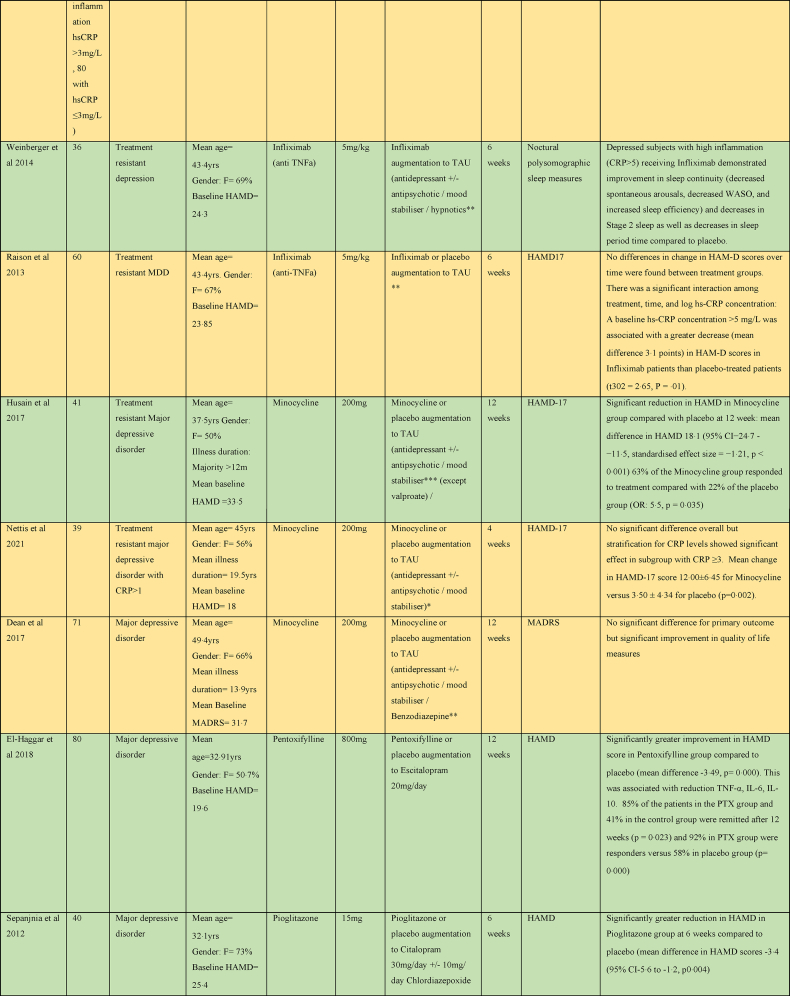

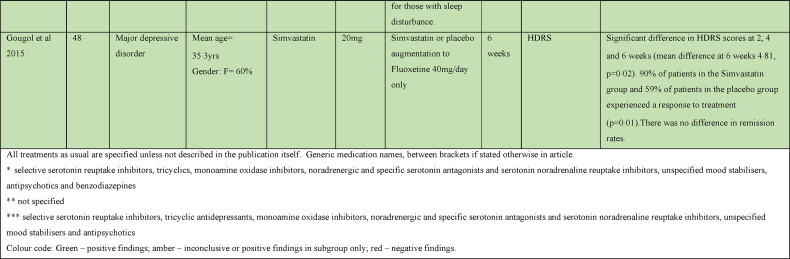
Summary of papers assessing anti-inflammatory medications in the treatment of major depressive disorder (alphabetically by medication).

#### 3.2.2 Acetylsalicylic acid (Aspirin)

Two studies using Aspirin in major depressive disorder as an adjunct to Duloxetine or Escitalopram did not show any significant difference between Aspirin and placebo HAMD scores at 6 months; however, the Aspirin + Duloxetine group showed quicker improvement in HAMD scores and better remission rate than the Escitalopram + placebo group (Zdanowicz et al., 2017). Another study providing augmentation of Aspirin with Citalopram had to be stopped prematurely because of adverse effects (severe anxiety and akathisia) (Ghanizadeh and Hedayati, 2014).

#### 3.2.3 Minocycline

Three studies examined the efficacy of Minocycline as an adjunct to TAU with medication (Husain et al., 2017; Dean et al., 2017; Nettis et al., 2021). One study found a significant benefit for Minocycline. This finding was not replicated in the other two studies (Dean et al., 2017), although one of them that only included patients with elevated levels of peripheral inflammation (defined as CRP≥1 mg/L) found some evidence of benefit for Minocycline in the high inflammation group after stratification based on CRP levels above or below 3 mg/L (Nettis et al., 2021)

#### 3.2.4 Cytokine inhibitors

Two studies examined the effect of cytokine inhibitors as an adjunct to TAU in major depression (Raison et al., 2013; Weinberger et al., 2015). One that examined the effect of Infliximab in 60 subjects with major depressive disorder did not find any benefit overall; but, found a greater response in HAMD score to Infliximab than placebo in subjects with evidence of inflammation (baseline hs-CRP concentration >5 mg/L) did have (Raison et al., 2013). A study of sleep parameters in a subset of this cohort found that subjects with high inflammation receiving Infliximab demonstrated improvement in sleep continuity and decreases in Stage 2 sleep (Weinberger et al., 2015).

#### 3.2.5 Other

Statins primarily lower cholesterol via inhibition of HMG-CoA reductase, but they are also thought to reduce cytokine release and reactive oxygen species generation via inhibition of the Rho and Rac signalling pathways, respectively. Statins have also been identified to upregulate endothelial nitric oxide synthase, an enzyme essential to nitric oxide (NO) production. NO plays an important role in maintaining endothelial homeostasis, regulating inflammatory states and inducing vasodilation (Antonopoulos et al., 2012).

A study evaluating Simvastatin augmentation of Fluoxetine in major depression found a significant improvement in HDRS scores and faster response rates in the Simvastatin group over placebo (Gougol et al., 2015). Another study found a significant benefit for Atorvastatin over placebo augmentation of Citalopram, found a significant benefit for Atorvastatin (Haghighi et al., 2014). Pioglitazone augmentation of Citalopram was beneficial with a significantly greater reduction in HAMD, earlier response to treatment and greater remission rates in the Pioglitazone group at 6 weeks compared to placebo (Sepanjnia et al., 2012).

Pentoxifylline evokes its anti-inflammatory affects by inhibition of phosphodiesterase-4. Phosphodiesterase-4 regulates cAMP within many pro-inflammatory of the immune system, and hence the production of several inflammatory cytokines, including IL-1, IL-6, interferon-ɣ and TNFα (Ghasemnejad-Berenji et al., 2021). A study evaluating Pentoxifylline augmentation of Escitalopram found a significantly greater improvement in HAMD score in the Pentoxifylline group (El-Haggar et al., 2018).

### 3.3 Schizophrenia and psychotic disorders

#### 3.3.1 Celecoxib

There were five studies assessing the efficacy of Celecoxib as an adjunct to antipsychotic treatment in schizophrenia, as shown in Table 4. Three studies reported a significant benefit on the PANSS with Celecoxib augmentation to Risperidone (Akhondzadeh et al., 2007; Müller et al., 2002; Zhang et al., 2021). One study of Celecoxib augmentation to Amisulpride did not find an effect on PANSS but did see an improvement in the secondary outcome clinical global impression (CGI) score (Muller et al., 2010). The remaining study did not report any benefit of Celecoxib augmentation to Risperidone or Olanzapine (Rapaport et al., 2005).

**Table 4:**
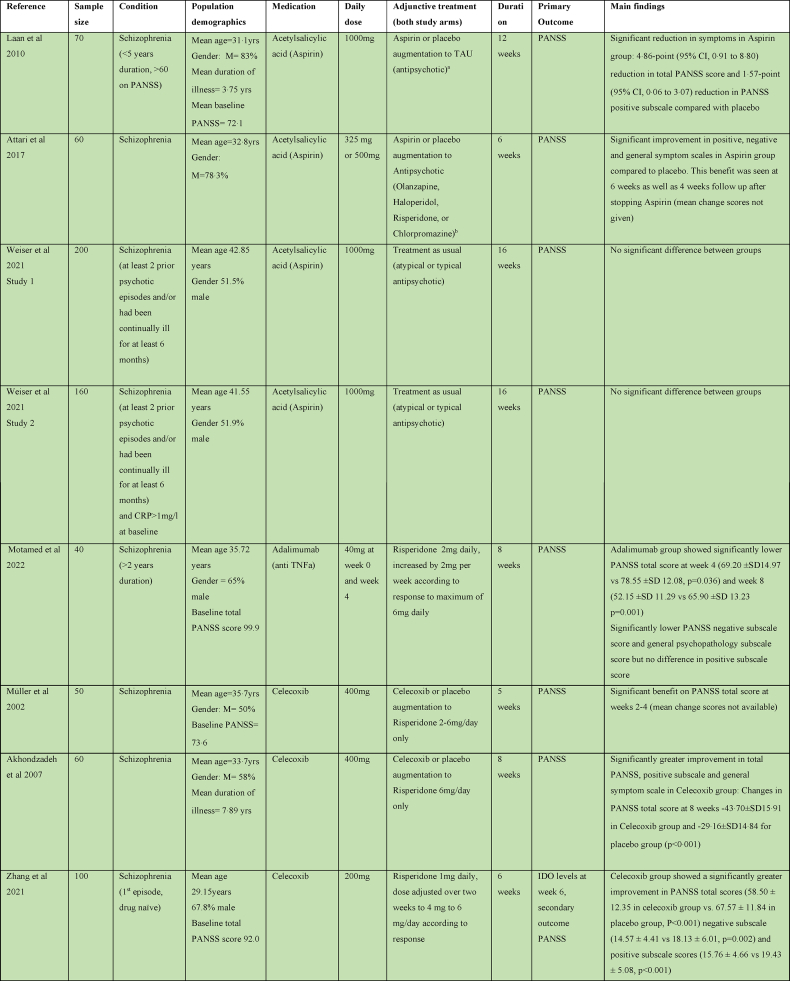

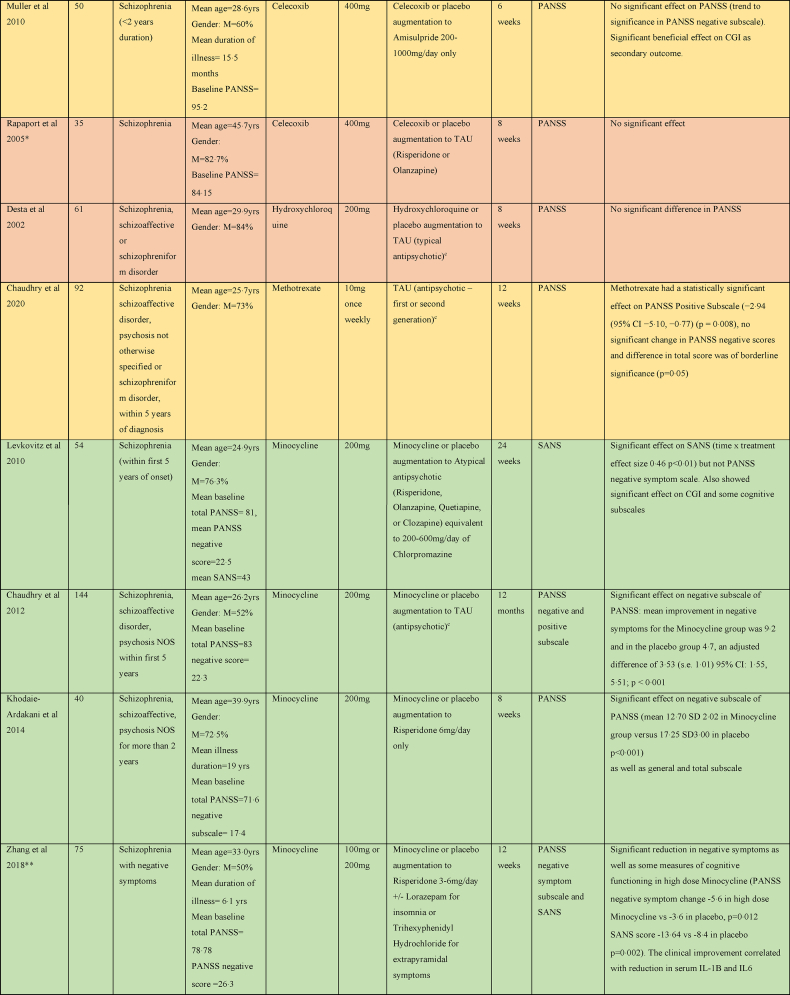

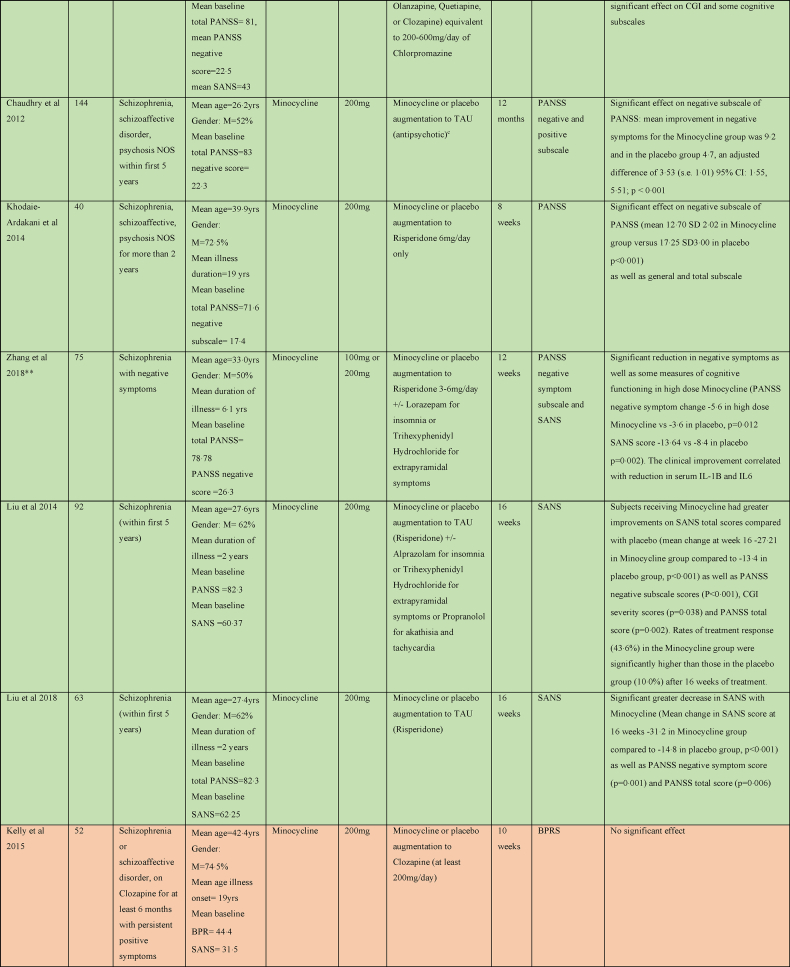

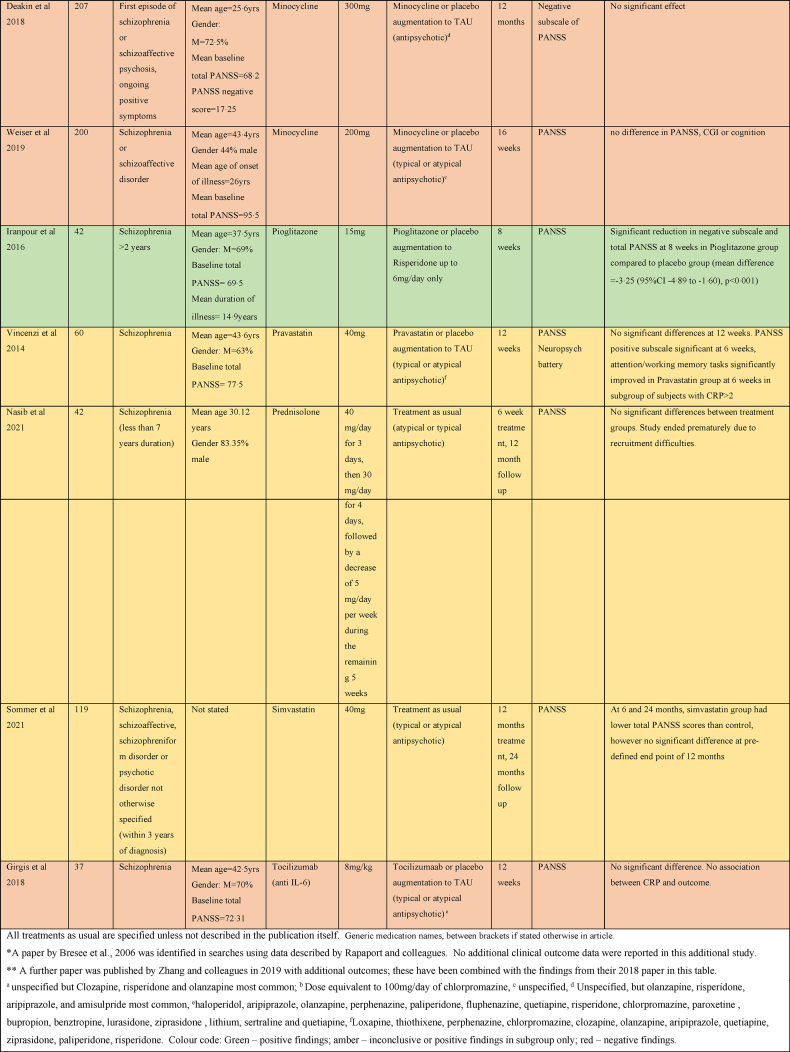
Summary of papers assessing anti-inflammatory medications in the treatment of Schizophrenia (alphabetically by medication).

#### 3.3.2 Acetylsalicylic acid (Aspirin)

Four studies considered the efficacy of Aspirin as an adjunct to usual antipsychotic treatment in schizophrenia. One study showed a significant reduction in total PANSS score and positive PANSS subscale compared with placebo at 12 weeks (Laan et al., 2010); one also showed reduction in negative and general PANSS subscales at 6 weeks (Attari et al., 2017). Two studies (reported in the same paper) did not show any benefit of Aspirin on PANSS by 16 weeks (Weiser et al., 2021).

#### 3.3.3 Minocycline

Six of nine studies exploring the efficacy of Minocycline in schizophrenia found benefit for negative symptoms (Levkovitz et al., 2010; Chaudhry et al., 2012; Zhang et al., 2018; Khodaie-Ardakani et al., 2014; Liu et al., 2014, 2018). One examined the effect of Minocycline augmentation to atypical antipsychotics on negative symptoms in early phase schizophrenia within the first 5 years of symptoms and found quicker response to treatment and a significant improvement on SANS, CGI, and executive functioning (Levkovitz et al., 2010). Another early phase study found effect of Minocycline added to TAU with medication on negative symptoms as a significant reduction in the PANSS negative subscale at 12 months (Chaudhry et al., 2012). Low dose (100 mg daily) and high dose (200 mg daily) Minocycline as an adjunct to Risperidone was found to effectuate a significant reduction in both SANS and PANSS negative symptom scales at 12 weeks (Zhang et al., 2018), and the clinical improvement correlated with reduction in serum IL-1B and IL6. A fourth study showed a quicker response to treatment and a significant reduction in PANSS negative subscale as well as total PANSS score with Minocycline augmentation of Risperidone (Khodaie-Ardakani et al., 2014). Two studies of Minocycline augmentation of Risperidone showed a significant decrease in SANS, PANSS negative symptom score and PANSS total score in the Minocycline group at 16 weeks (Liu et al., 2014, 2018).

Three studies did not show any benefit on psychotic symptoms of Minocycline in the treatment of schizophrenia (Kelly et al., 2015; Weiser et al., 2019; Deakin et al., 2018). Two were multicentre trials comparing Minocycline augmentation of usual antipsychotic treatment in 200 and 207 participants respectively (Weiser et al., 2019; Deakin et al., 2018). However, a third study that looked at Minocycline as an adjunct to Clozapine did not shown improvement of psychotic symptoms, but reported significant improvements in depressive, anxiety and cognitive symptoms (Kelly et al., 2015).

#### 3.3.4 Cytokine inhibitors

One study examining Tocilizumab, an IL6 receptor antagonist, in the treatment of schizophrenia as an adjunct to usual antipsychotic treatment did not find any significant difference in the PANSS at 12 weeks (Girgis et al., 2018). One study examined Adalimumab, a TNF-α inhibitor, as an adjunctive treatment to Risperidone, and did find significantly lower total PANSS scores, negative subscale score and general psychopathology subscale scores in the Adalimumab group at 8 weeks (Motamed et al., 2022).

#### 3.3.5 Other

Other medications trialled in schizophrenia were Pravastatin, Simvastatin, Pioglitazone, Methotrexate, Hydroxychloroquine and Prednisolone. A trial of Pravastatin augmentation of TAU did not show any significant benefit in outcomes except for a significant decrease in the PANSS positive symptoms score at 6 weeks; however, this was not seen at the 12-week study endpoint (Vincenzi et al., 2014). Simvastatin augmentation of TAU resulted in lower total PANSS scores in the Simvastatin group at 6 and 24 months; however, there were no significant difference at pre-defined end point of 12 months (Sommer et al., 2021). A trial of Pioglitazone as an adjunct to Risperidone showed a significant reduction in negative subscale and total PANSS at 8 weeks in the Pioglitazone group compared to placebo (Iranpour et al., 2016).

Methotrexate has been shown to inhibit NF-κB activation, increase T-cell sensitivity to apoptosis and increase extracellular adenosine which binds to cell surface receptors to prevent pro-inflammatory signalling (Cronstein and Aune, 2020). A recent study of Methotrexate as an adjunct to TAU had a statistically significant effect on PANSS Positive Subscale but no significant effect on negative subscale and overall, the difference in total PANSS score was of borderline significance (Chaudhry et al., 2020). Hydroxychloroquine induces its anti-inflammatory affects by inhibiting activation of the toll-like-receptor-9, a receptor that triggers a pro-inflammatory response to microbial products (Kužnik et al., 2011). The study of Hydroxychloroquine as an adjunct to typical antipsychotic treatment in 61 participants showed no significant benefit (Desta et al., 2002). The study of Prednisolone augmentation of TAU did not show any benefit at 6 weeks or 12 months (Nasib et al., 2021).

### 3.4 SSRD: chronic fatigue syndrome

Only one study was found relating to SSRD as shown in Table 5. This study assessed the efficacy of Anakinra (an interleukin-1 antagonist) in 50 women with chronic fatigue syndrome. This was not an augmentation study as currently no pharmacological treatment for chronic fatigue syndrome exists. It did not find a clinically significant reduction in fatigue severity with Anakinra (Roerink et al., 2017).

**Table 5:**
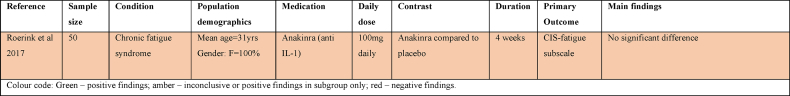
Summary of papers assessing anti-inflammatory medications in the treatment of Somatic Symptom Related Disorders.

### 3.5 Adverse events

Across all studies there was no difference in the incidence of serious drug side effects requiring hospitalisation, except in one study. The study evaluating Aspirin or placebo augmentation to Citalopram found that eight out of ten patients showed severe anxiety and akathisia from the early days of this trial, that necessitated discontinuation of the medication and hospitalization of three patients. Also, two patients reported suicidal behaviour after the onset of this trial (Ghanizadeh and Hedayati, 2014).

Only two studies reported a significant increase in side effects within the Celecoxib vs placebo group (Nery et al., 2008; Muller et al., 2006). Muller et al. (2006) reported 4 patients of 20 that developed hypertension, sleep-disturbance, difficulties in miction or erection and rash within the Celecoxib group for treatment of MDD (Muller et al., 2006). Nery et al. (2008) also reported that 2 of 14 patients within the Celecoxib group for treatment of bipolar depression developed rash (Nery et al., 2008). No studies reported an increased incidence of gastrointestinal side effects within the Celecoxib group.

## 4 Discussion

### 4.1 Summary of the findings

This scoping review finds evidence for effect of anti-inflammatory drugs across a wide range of medications and mental disorders. Since the 2014 review (Fond et al., 2014), in this rapidly evolving field there have been 37 further trials published, making this paper an important update. It has shown that there is now a large body of evidence examining the use of a variety of anti-inflammatory medication in bipolar affective disorder, major depressive disorder and schizophrenia. However, the use of such medication in SSRD is not well researched. The results show that all but one study (Roerink et al., 2017) evaluate augmentation to other treatment and that there is heterogeneity in terms of the treatment to which an anti-inflammatory medication is augmented.

### 4.2 Mood disorders

Regarding bipolar disorder, the studies evaluating augmentation with Celecoxib to Sodium Valproate or to Escitalopram, or Pioglitazone augmentation to Lithium only, show significant improvement. However, studies evaluating augmentation with other anti-inflammatory medication such as Aspirin, Minocycline or Infliximab on usual treatment show negative or inconclusive results.

This might be explained by different disease profiles. For example, the presence or absence of psychotic symptoms and acuity of disease may affect the choice of medication. Also, the choice of mood stabiliser can be associated with illness phase, for example some medications are provided in early stages of the illness, and others are second- or third-line medications. Allowing all available medications in TAU therefore neglects acuity of the illness, which should be taken into account. Furthermore, patients may have had an adverse reaction to a particular mood stabiliser and therefore receive another one, which may be related to their genetic profile. These factors may cause ambiguous results when anti-inflammatory agents are augmented to miscellaneous medications.

A variety of other medications were evaluated in several rather small studies. Aspirin seemed effective if combined with N-Acetylcysteine or Minocycline, but not as a standalone treatment. Minocycline was effective especially if provided to patients with elevated inflammation markers. One study exploring Infliximab in case of elevated inflammation markers found effect in a participants with a history of childhood maltreatment only (McIntyre et al., 2019). To control for such variety to enable comparison, studies are needed augmenting these anti-inflammatory medications to single psychotropic medications such as Escitalopram, Sodium Valproate or Lithium only.

Currently, there is evidence for the effect of Celecoxib or Pioglitazone augmentation in bipolar disorder, but no firm conclusion can be drawn regarding the other anti-inflammatory medications that were evaluated.

Similarly, in major depressive disorder, the majority of studies found benefit for Celecoxib augmentation of SSRI treatment. A few trials reported effect of Minocycline in treatment resistant depression, especially for those participants with elevated inflammation markers. Infliximab was also found to be effective in case of elevated inflammation markers. Trials evaluating augmentation with Simvastatin, Atorvastatin, Pioglitazone and Pentoxifylline to Escitalopram or Fluoxetine all showed effect. This supports evidence from large scale observational studies which have found lower rates of depression in patients taking statins (Redlich et al., 2014). In general, it is striking that studies augmenting monotreatment with SSRIs such as Escitalopram, Sertraline or Fluoxetine with anti-inflammatory medication in mood disorders have shown benefit. This may indicate a synergism in mechanism between the anti-inflammatory medication and the SSRI.

### 4.3 Schizophrenia

The findings of studies examining Celecoxib in Schizophrenia appear more mixed; studies augmenting Risperidone with Celecoxib show significant improvement in young patients with schizophrenia; however, a study evaluating Celecoxib in patients receiving Risperidone or Olanzapine, of higher average age, was inconclusive. Likewise, studies looking at Aspirin in schizophrenia have shown benefit in young patients, but not in patients with average age over 30 years. Such an age effect does not apply for Adalimumab, suggesting that age may be relevant in effect of medication for vascular inflammation like Celecoxib and Aspirin, but not in a TNFα blocker or antibiotics.

Studies evaluating Simvastatin and Prednisolone in schizophrenia showed no improvement.

Minocycline appeared to have a beneficial effect on negative symptoms in schizophrenia in the majority of early studies. However, the two most recent studies (Weiser et al., 2019; Deakin et al., 2018) did not see any benefit. These studies were well designed with larger sample sizes than previously; however, there was no evidence of inflammation within the study populations and the authors highlight that the longer duration of illness may have meant that any putative neuroinflammation had ceased by the time of participation in the study, as discussed below.

The effect of Minocycline augmentation in schizophrenia is more convincing than in other disorders and this might suggest a different underlying mechanism for treatment success than in the other main illness groups. This effect appears especially in young patients. These findings warrant further exploration of different anti-inflammatory treatment pathways for schizophrenia and mood disorder.

### 4.4 Therapeutic mechanisms

The mechanism in which augmentation with anti-inflammatory agents to regular psychiatric mediation improves psychiatric symptoms remains largely uncertain. Multiple neuro-inflammatory pathways have been implicated in depression (Woelfer et al., 2019), bipolar disorder (Benedetti et al., 2020) and schizophrenia (Müller et al., 2015). It may be that adding an anti-inflammatory offers an approach to improve the mental disorder from two ends: by decreasing the inflammation that helped generate the disorder, and by directly improving the symptoms that come from it. Moreover, psychotropic medications may exert an effect on inflammation (Sugino et al., 2009; Al-Amin et al., 2013; Hamer et al., 2011; Basterzi et al., 2005; Giridharan et al., 2020) and this may vary between drug classes; therefore, synergistic mechanisms may occur. This is supported by the finding that most of the studies that stratified by inflammatory markers found better efficacy in those patients who had evidence of inflammation at baseline: Nettis et al. found an effect for Minocycline in depression only in the subgroup of patients with CRP>/3 (Nettis et al., 2021). Higher baseline IL-6 levels also predicted response. Similarly, Savitz et al. found patients with bipolar depression with higher IL-6 levels at baseline responded better to Minocycline (Savitz et al., 2018). Raison and colleagues observed improvement with Infliximab therapy only in depressed patients with an hsCRP >5 mg/l (Raison et al., 2013) Further research is needed to explore this.

The medications included in this review target both upstream and downstream inflammatory pathways. The Cox-2 inhibitor, Celecoxib is an example of a downstream inhibitor that has shown particular therapeutic promise in this review. This might suggest that the prostaglandin pathway plays an important role in therapeutic mechanisms of anti-inflammatories in psychiatric disease, especially major depressive disorder (Chu et al., 2017).

Regarding the clear advantage of augmentation with Minocycline in psychosis, this may in a similar way act on the CNS inflammation by improving microglia function; but also, it may be that the effect of Minocycline is related to antibiotic effects on an as yet unidentified bacteria that plays a role in the development or the persistence of schizophrenia-like psychotic symptoms. Although epidemiological associations between schizophrenia and exposure to infectious agents including Toxoplasmosis and cat scratch disease have been found, a causal link has remained elusive (Endres et al., 2020, 2022; Fan and Ali, 2020; Fuglewicz et al., 2017). There may also be effects on translocation of bacteria across the gut wall, thus influencing the brain-gut axis (Nemani et al., 2015). The positive effect of Minocycline warrants further research into the mechanism.

The effect of cytokine inhibitors may be result of a direct effect on pro-inflammatory cytokines, and further research is needed to explore this. For example, a study on Conversion Disorder/Functional Neurological Disorder (CD/FND), a subclassification of SSRD, found several elevated inflammation markers in FND patients, however IL-1b levels were normal (van der Feltz-Cornelis et al., 2021). This may be relevant given the finding that an IL1-b blocker was ineffective in treatment of chronic fatigue syndrome (Redlich et al., 2014). Chronic fatigue occurs often in FND, and maybe there are similarities in terms of underlying mechanism that would be relevant to the choice of anti-inflammatory medication in future research.

Finally, age and duration of illness also may play a role, especially in schizophrenia, where anti-inflammatory drugs working on vascular inflammation mostly seem to be effective in patients younger than 30. This suggests that the role of vascular inflammation may become less outspoken as a driver in long-term schizophrenia, but other inflammatory mechanisms may still be a relevant process at higher age.

### 4.5 Strengths and limitations

In this article we sought to only review higher level evidence, hence we selected only RCTs. Consequently, many case reports and cohort studies were excluded. These included studies assessing the role of anti-inflammatory medications in SSRD. Also, since we excluded cohorts with major somatic comorbidities, a trial establishing the psychotropic benefits of Metformin, an antidiabetic medicine, was excluded (Guo et al., 2014). Furthermore, 13 articles were excluded as authors were unable to gain full access to the text.

The included studies were highly heterogenous in terms of their population demographics, illness stage, duration and severity, and adjunctive psychotropic medication used. They did not take comorbid mental disorders into account, which may explain some of the variation in findings.

When measuring symptomatology, a variety of different tools were used. Many studies failed to control for baseline depression or PANNS scores by not reporting the change in scores (Higgins et al., 2021). Most studies were rather small, and the studies with more participants all reported inconclusive results. However, those larger studies did not take level of inflammation into account in the analysis. Most studies did not stratify for biomarkers of inflammation at baseline.

### 4.6 Implications for further research

Future studies need to be targeted at patients with evidence of inflammation; however, it should be noted that peripheral inflammatory markers such as CRP can fluctuate, and are affected by confounding factors such as obesity and smoking (Yudkin et al., 1999). Future studies should also take comorbidity into account, both with somatic conditions, such as for example diabetes with comorbid depressive disorder (van der Feltz‐Cornelis et al., 2021), and comorbid mental disorders, such as for example psychosis and mood disorder. This might have implications for treatment effect of a particular drug, so study participants should be well described in terms of comorbidity; if possible, selection should be done avoiding such comorbidity.

In general, higher quality studies are needed. In particular, baseline symptom scores should be controlled for in analyses by calculating change scores. Studies should specify the stage of illness (chronic vs acute) in their cohort and, if possible, augment to monotreatment only instead to a variety of TAU medications. Longer term studies are required to assess optimum treatment duration and dose, as well as the incidence of long-term adverse events.

## 5 Conclusion

The findings of this review suggest that there is scope to consider the use of anti-inflammatory agents in mental disorders; however, not as a one-size-fits-all solution. Treatment could be helpful in case of baseline inflammation. Anti-inflammatory medications that seem mostly effective in bipolar disorder or major depressive disorder, such as Celecoxib, Pioglitazone and statins, may differ from the ones with indications of effectiveness in schizophrenia, such as Minocycline and Aspirin. This might suggest a different underlying mechanism for treatment success in those two main illness groups. Further studies with larger sample sizes that take inflammation markers into account are needed to confirm these findings. The medications reviewed were found to be well tolerated, except, in one trial, Aspirin. Research into the possible role of anti-inflammatory medication in SSRD is at an early stage with limited evidence and requires further exploration.

## Author statement

The concept for this work was developed by CFC, JS, RF and WH-C. JS and RF developed the search strategy under the supervision of CFC. Searches were run by JS and RF. All authors contributed to hand searching. Title, abstract and full text screening was undertaken by RF and JS with discrepancies resolved through discussion with CFC. RF and WH-C completed additional full-text screening to identify studies using medications of interest for anti-inflammatory purposes. RF, JS and WH-C completed data extraction under the supervision of CFC. RF, JS and WHC contributed to early drafts of the manuscript. CFC wrote the final version of the manuscript and all authors have reviewed and edited the final manuscript.

## Funding

The authors received no financial support for the research, authorship, and/or publication of this article.

## Declaration of competing interest

The authors declare that they have no known competing financial interests or personal relationships that could have appeared to influence the work reported in this paper.

## Data Availability

No data was used for the research described in the article.

## References

[bib1] Abbasi S.H., Hosseini F., Modabbernia A., Ashrafi M., Akhondzadeh S. (2012). Effect of celecoxib add-on treatment on symptoms and serum IL-6 concentrations in patients with major depressive disorder: randomized double-blind placebo-controlled study. J. Affect. Disord..

[bib2] Akhondzadeh S., Tabatabaee M., Amini H., Ahmadi Abhari S.A., Abbasi S.H., Behnam B. (2007). Celecoxib as adjunctive therapy in schizophrenia: a double-blind, randomized and placebo-controlled trial. Schizophr. Res..

[bib3] Akhondzadeh S., Jafari S., Raisi F., Nasehi A.A., Ghoreishi A., Salehi B. (2009). Clinical trial of adjunctive celecoxib treatment in patients with major depression: a double blind and placebo controlled trial. Depress. Anxiety.

[bib4] Al-Amin M.M., Uddin M.M.N., Reza H.M. (2013). Effects of antipsychotics on the inflammatory response system of patients with schizophrenia in peripheral blood mononuclear cell cultures. Clin. Psychopharmacol. Neurosci..

[bib5] Antonopoulos A.S., Margaritis M., Lee R., Channon K., Antoniades C. (2012 Apr 1). Statins as anti-inflammatory agents in atherogenesis: molecular mechanisms and lessons from the recent clinical trials. Curr. Pharmaceut. Des..

[bib6] Arabzadeh S., Ameli N., Zeinoddini A., Rezaei F., Farokhnia M., Mohammadinejad P. (2015). Celecoxib adjunctive therapy for acute bipolar mania: a randomized, double-blind, placebo-controlled trial. Bipolar Disord..

[bib7] Attari A., Mojdeh A., Khalifeh Soltani F.A.S., Najarzadegan M.R. (2017). Aspirin inclusion in antipsychotic treatment on severity of symptoms in schizophrenia: a randimized clinical trial. Iranian J. Psychiatr. Behav. Sci..

[bib8] Basterzi A.D., Aydemir Ç., Kisa C., Aksaray S., Tuzer V., Yazici K. (2005). IL‐6 levels decrease with SSRI treatment in patients with major depression. Hum. Psychopharmacol. Clin. Exp..

[bib9] Bauer I.E., Green C., Colpo G.D., Teixeira A.L., Selvaraj S., Durkin K. (2018). A double-blind, randomized, placebo-controlled study of aspirin and N-acetylcysteine as adjunctive treatments for bipolar depression. J. Clin. Psychiatr..

[bib10] Baune B.T., Sampson E., Louise J., Hori H., Schubert K.O., Clark S.R., Mills N.T., Fourrier C. (2021 Dec 1). No evidence for clinical efficacy of adjunctive celecoxib with vortioxetine in the treatment of depression: a 6-week double-blind placebo controlled randomized trial. Eur. Neuropsychopharmacol.

[bib11] Benedetti F., Aggio V., Pratesi M.L., Greco G., Furlan R. (2020 Feb 26). Neuroinflammation in bipolar depression. Front. Psychiatr..

[bib12] Chaudhry I.B., Hallak J., Husain N., Minhas F., Stirling J., Richardson P. (2012). Minocycline benefits negative symptoms in early schizophrenia: a randomised double-blind placebo-controlled clinical trial in patients on standard treatment. J. Psychopharmacol..

[bib13] Chaudhry I.B., Husain M.O., Khoso A.B., Husain M.I., Buch M.H., Kiran T. (2020). A randomised clinical trial of methotrexate points to possible efficacy and adaptive immune dysfunction in psychosis. Transl. Psychiatry.

[bib14] Chu C., Wei H., Zhu W., Shen Y., Xu Q. (2017 Sep). Decreased prostaglandin D2 levels in major depressive disorder are associated with depression-like behaviors. Int. J. Neuropsychopharmacol..

[bib15] Cronstein B.N., Aune T.M. (2020 Mar). Methotrexate and its mechanisms of action in inflammatory arthritis. Nat. Rev. Rheumatol..

[bib16] Dantzer R. (2009). Cytokine, sickness behavior, and depression. Immunol. Allergy Clin..

[bib17] Deakin B., Suckling J., Barnes T.R.E., Byrne K., Chaudhry I.B., Dazzan P. (2018). The benefit of minocycline on negative symptoms of schizophrenia in patients with recent-onset psychosis (BeneMin): a randomised, double-blind, placebo-controlled trial. Lancet Psychiatr..

[bib18] Dean O.M., Kanchanatawan B., Ashton M., Mohebbi M., Ng C.H., Maes M. (2017). Adjunctive minocycline treatment for major depressive disorder: a proof of concept trial. Aust. N. Z. J. Psychiatr..

[bib19] Desta M.T.A., Gebre N., Barci B.M., Fuller Torrey E., Knable M. (2002). Controlled trial of hydroxychloroquine in schizophrenia. J. Clin. Psychopharmacol..

[bib20] Edberg D., Hoppensteadt D., Walborn A., Fareed J., Sinacore J., Halaris A. (2018). Plasma C-reactive protein levels in bipolar depression during cyclooxygenase-2 inhibitor combination treatment. J. Psychiatr. Res..

[bib21] El-Haggar S.M., Eissa M.A., Mostafa T.M., El-Attar K.S., Abdallah M.S. (2018). The phosphodiesterase inhibitor pentoxifylline as a novel adjunct to antidepressants in major depressive disorder patients: a proof-of-concept, randomized, double-blind, placebo-controlled trial. Psychother. Psychosom..

[bib22] Elewa H.F., Hilali H., Hess D.C., Machado L.S., Fagan S.C. (2006 Apr). Minocycline for short‐term neuroprotection. Pharmacotherapy.

[bib23] Enache D., Nikkheslat N., Fathalla D., Morgan B.P., Lewis S., Drake R. (2021). Peripheral immune markers and antipsychotic non-response in psychosis. Schizophr. Res..

[bib24] Endres D., Leypoldt F., Bechter K., Hasan A., Steiner J., Domschke K., Wandinger K.P., Falkai P., Arolt V., Stich O., Rauer S. (2020 Oct). Autoimmune encephalitis as a differential diagnosis of schizophreniform psychosis: clinical symptomatology, pathophysiology, diagnostic approach, and therapeutic considerations. Eur. Arch. Psychiatr. Clin. Neurosci..

[bib25] Endres D., Maier V., Leypoldt F., Wandinger K.P., Lennox B., Pollak T.A., Nickel K., Maier S., Feige B., Domschke K., Prüss H. (2022 Apr). Autoantibody-associated psychiatric syndromes: a systematic literature review resulting in 145 cases. Psychol. Med..

[bib26] Fan J., Ali H. (2020 Jul 2). Cat scratch disease causing encephalitis. InBaylor Univ. Med. Center. Proc..

[bib27] van der Feltz-Cornelis C.M., Bakker M., Kaul A., Kuijpers T.W., von Känel R. (2020). IL-6 and hsCRP in Somatic Symptom Disorders and related disorders. Brain. Behav. Immun.Health..

[bib28] van der Feltz-Cornelis C., Brabyn S., Ratcliff J., Varley D., Allgar V., Gilbody S. (2021). Assessment of cytokines, microRNA and patient related outcome measures in conversion disorder/functional neurological disorder (CD/FND): the CANDO clinical feasibility study. Brain. Behav. Immun.Health..

[bib29] van der Feltz‐Cornelis C., Allen S.F., Holt R.I., Roberts R., Nouwen A., Sartorius N. (2021). Treatment for comorbid depressive disorder or subthreshold depression in diabetes mellitus: systematic review and meta‐analysis. Brain Behav..

[bib30] Fond G., Hamdani N., Kapczinski F., Boukouaci W., Drancourt N., Dargel A. (2014). Effectiveness and tolerance of anti‐inflammatory drugs' add‐on therapy in major mental disorders: a systematic qualitative review. Acta Psychiatr. Scand..

[bib31] Frydecka D., Krzystek-Korpacka M., Lubeiro A., Stramecki F., Stańczykiewicz B., Beszłej J.A. (2018). Profiling inflammatory signatures of schizophrenia: a cross-sectional and meta-analysis study. Brain Behav. Immun..

[bib32] Fuglewicz A.J., Piotrowski P., Stodolak A. (2017 Sep 1). Relationship between toxoplasmosis and schizophrenia: a review. Adv. Clin. Exp. Med..

[bib33] Ghanizadeh A., Hedayati A. (2014). Augmentation of citalopram with aspirin for treating major depressive disorder, a double blind randomized placebo controlled clinical trial. Anti-Inflammatory Anti-Allergy Agents Med. Chem..

[bib34] Ghasemnejad-Berenji M., Pashapour S., Sadeghpour S. (2021). Pentoxifylline: a drug with antiviral and anti-inflammatory effects to be considered in the treatment of coronavirus disease 2019. Med. Princ. Pract..

[bib35] Girgis R.R., Ciarleglio A., Choo T., Haynes G., Bathon J.M., Cremers S. (2018). A randomized, double-blind, placebo-controlled clinical trial of Tocilizumab, an interleukin-6 receptor antibody, for residual symptoms in schizophrenia. Neuropsychopharmacology.

[bib36] Giridharan V., Scaini G., Colpo G.D., Doifode T., F Pinjari O., Teixeira A.L. (2020). Clozapine prevents poly (I: C) induced inflammation by modulating NLRP3 pathway in microglial cells. Cells.

[bib37] Goldsmith D., Rapaport M., Miller B. (2016). A meta-analysis of blood cytokine network alterations in psychiatric patients: comparisons between schizophrenia, bipolar disorder and depression. Mol. Psychiatr..

[bib38] Gougol A., Zareh-Mohammadi N., Raheb S., Farokhnia M., Salimi S., Iranpour N. (2015). Simvastatin as an adjuvant therapy to fluoxetine in patients with moderate to severe major depression: a double-blind placebo-controlled trial. J. Psychopharmacol..

[bib39] Guo M., Mi J., Jiang Q.M., Xu J.M., Tang Y.Y., Tian G. (2014). Metformin may produce antidepressant effects through improvement of cognitive function among depressed patients with diabetes mellitus. Clin. Exp. Pharmacol. Physiol..

[bib40] Haapakoski R., Mathieu J., Ebmeier K.P., Alenius H., Kivimäki M. (2015). Cumulative meta-analysis of interleukins 6 and 1β, tumour necrosis factor α and C-reactive protein in patients with major depressive disorder. Brain Behav. Immun..

[bib41] Haghighi M., Khodakarami S., Jahangard L., Ahmadpanah M., Bajoghli H., Holsboer-Trachsler E. (2014). In a randomized, double-blind clinical trial, adjuvant atorvastatin improved symptoms of depression and blood lipid values in patients suffering from severe major depressive disorder. J. Psychiatr. Res..

[bib42] Halaris A., Cantos A., Johnson K., Hakimi M., Sinacore J. (2020). Modulation of the inflammatory response benefits treatment-resistant bipolar depression: a randomized clinical trial. J. Affect. Disord..

[bib43] Hamer M., Batty G.D., Marmot M.G., Singh-Manoux A., Kivimäki M. (2011). Anti-depressant medication use and C-reactive protein: results from two population-based studies. Brain Behav. Immun..

[bib44] Higgins J., Thomas J., Chandler J., Cumpston M., Li T., Page M. (2021). Cochrane handbook for systematic reviews of interventions version 6.2 (updated february 2021): Cochrane. http://www.training.cochrane.org/handbook.

[bib45] Houtveen J.H., Kavelaars A., Heijnen C.J., van Doornen L.J. (2007). Heterogeneous medically unexplained symptoms and immune function. Brain Behav. Immun..

[bib46] Howren M.B., Lamkin D.M., Suls J. (2009). Associations of depression with C-reactive protein, IL-1, and IL-6: a meta-analysis. Psychosom. Med..

[bib47] Husain M.I., Chaudhry I.B., Husain N., Khoso A.B., Rahman R.R., Hamirani M.M. (2017). Minocycline as an adjunct for treatment-resistant depressive symptoms: a pilot randomised placebo-controlled trial. J. Psychopharmacol..

[bib48] Husain M.I., Chaudhry I.B., Khoso A.B., Husain M.O., Hodsoll J., Ansari M.A. (2020). Minocycline and celecoxib as adjunctive treatments for bipolar depression: a multicentre, factorial design randomised controlled trial. Lancet Psychiatr..

[bib49] Iranpour N., Zandifar A., Farokhnia M., Goguol A., Yekehtaz H., Khodaie-Ardakani M.R. (2016). The effects of pioglitazone adjuvant therapy on negative symptoms of patients with chronic schizophrenia: a double-blind and placebo-controlled trial. Hum. Psychopharmacol..

[bib50] Kaplan J., Nowell M., Chima R., Zingarelli B. (2014 Jul). Pioglitazone reduces inflammation through inhibition of NF-κB in polymicrobial sepsis. Innate Immun..

[bib51] Kappelmann N., Lewis G., Dantzer R., Jones P., Khandaker G. (2018). Antidepressant activity of anti-cytokine treatment: a systematic review and meta-analysis of clinical trials of chronic inflammatory conditions. Mol. Psychiatr..

[bib52] Kelly D.L., Sullivan K.M., McEvoy J.P., McMahon R.P., Wehring H.J., Gold J.M. (2015). Adjunctive minocycline in clozapine-treated schizophrenia patients with persistent symptoms. J. Clin. Psychopharmacol..

[bib53] Khodaie-Ardakani M.R., Mirshafiee O., Farokhnia M., Tajdini M., Hosseini S.M., Modabbernia A. (2014). Minocycline add-on to risperidone for treatment of negative symptoms in patients with stable schizophrenia: randomized double-blind placebo-controlled study. Psychiatr. Res..

[bib54] Kose M., Pariante C.M., Dazzan P., Mondelli V. (2021). The role of peripheral inflammation in clinical outcome and brain imaging abnormalities in psychosis: a systematic review. Front. Psychiatr..

[bib55] Kužnik A., Benčina M., Švajger U., Jeras M., Rozman B., Jerala R. (2011 Apr 15). Mechanism of endosomal TLR inhibition by antimalarial drugs and imidazoquinolines. J. Immunol..

[bib56] Laan W., Grobbee D.E., Selten J.P., Heijnen C.J., Kahn R.S., Burger H. (2010). Adjuvant aspirin therapy reduces symptoms of schizophrenia spectrum disorders: results from a randomized, double-blind, placebo-controlled trial. J. Clin. Psychiatr..

[bib57] Levkovitz Y., Mendlovich S., Riwkes S., Braw Y., Levkovitch-Verbin H., Gal G. (2010). A double-blind, randomized study of minocycline for the treatment of negative and cognitive symptoms in early-phase schizophrenia. J. Clin. Psychiatr..

[bib58] Liu F., Guo X., Wu R., Ou J., Zheng Y., Zhang B. (2014). Minocycline supplementation for treatment of negative symptoms in early-phase schizophrenia: a double blind, randomized, controlled trial. Schizophr. Res..

[bib59] Liu T., Zhang L., Joo D., Sun S.C. (2017 Jul 14). NF-κB signaling in inflammation. Signal Transduct. Targeted Ther..

[bib60] Liu F., Zhang B., Xie L., Ruan Y., Xu X., Zeng Y. (2018). Changes in plasma levels of nitric oxide metabolites and negative symptoms after 16-week minocycline treatment in patients with schizophrenia. Schizophr. Res..

[bib61] Majd M.H.F., Hosseini M., Shariatpanahic V., Sharifi A. (2015). A randomized, double-blind, placebo-controlled trial of celecoxib augmentation of sertraline in treatment of drug-naive depressed women: a pilot study. Iran. J. Pharm. Res. (IJPR).

[bib62] McHugh M.L. (2012). Interrater reliability: the kappa statistic. Biochem. Med..

[bib63] McIntyre R.S., Subramaniapillai M., Lee Y., Pan Z., Carmona N.E., Shekotikhina M. (2019). Efficacy of adjunctive infliximab vs placebo in the treatment of adults with bipolar I/II depression: a randomized clinical trial. JAMA Psychiatr..

[bib64] Miller B.J., Buckley P., Seabolt W., Mellor A., Kirkpatrick B. (2011). Meta-analysis of cytokine alterations in schizophrenia: clinical status and antipsychotic effects. Biol. Psychiatr..

[bib65] Mondelli V., Ciufolini S., Belvederi Murri M., Bonaccorso S., Di Forti M., Giordano A. (2015). Cortisol and inflammatory biomarkers predict poor treatment response in first episode psychosis. Schizophr. Bull..

[bib66] Motamed M., Karimi H., Sanjari Moghaddam H., Taherzadeh Boroujeni S., Sanatian Z., Hasanzadeh A., Khodaei Ardakani M.R., Akhondzadeh S. (2022 Mar 31). Risperidone combination therapy with adalimumab for treatment of chronic schizophrenia: a randomized, double-blind, placebo-controlled clinical trial. Int. Clin. Psychopharmacol..

[bib67] Müller N., Riedel M., Scheppach C., Brandstätter B., Sokullu S., Krampe K., Ulmschneider M., Engel R.R., Möller H.J., Schwarz M.J. (2002). Beneficial antipsychotic effects of celecoxib add-on therapy compared to risperidone alone in schizophrenia. Am. J. Psychiatr..

[bib68] Muller N., Schwarz M.J., Dehning S., Douhe A., Cerovecki A., Goldstein-Muller B. (2006). The cyclooxygenase-2 inhibitor celecoxib has therapeutic effects in major depression: results of a double-blind, randomized, placebo controlled, add-on pilot study to reboxetine. Mol. Psychiatr..

[bib69] Muller N., Krause D., Dehning S., Musil R., Schennach-Wolff R., Obermeier M. (2010). Celecoxib treatment in an early stage of schizophrenia: results of a randomized, double-blind, placebo-controlled trial of celecoxib augmentation of amisulpride treatment. Schizophr. Res..

[bib70] Müller N., Weidinger E., Leitner B., Schwarz M.J. (2015 Oct 21). The role of inflammation in schizophrenia. Front. Neurosci..

[bib71] Munn Z., Peters M.D., Stern C., Tufanaru C., McArthur A., Aromataris E. (2018). Systematic review or scoping review? Guidance for authors when choosing between a systematic or scoping review approach. BMC Med. Res. Methodol..

[bib72] Nasib L.G., Gangadin S.S., Winter-van Rossum I., Boudewijns Z.S., de Witte L.D., Wilting I., Luykx J., Somers M., Veen N., van Baal C., Kahn R.S. (2021 Apr 1). The effect of prednisolone on symptom severity in schizophrenia: a placebo-controlled, randomized controlled trial. Schizophr. Res..

[bib73] Nemani K., Ghomi R.H., McCormick B., Fan X. (2015 Jan 2). Schizophrenia and the gut–brain axis. Prog. Neuro Psychopharmacol. Biol. Psychiatr..

[bib74] Nery F.G., Monkul E.S., Hatch J.P., Fonseca M., Zunta-Soares G.B., Frey B.N. (2008). Celecoxib as an adjunct in the treatment of depressive or mixed episodes of bipolar disorder: a double-blind, randomized, placebo-controlled study. Hum. Psychopharmacol..

[bib75] Nettis M.A., Lombardo G., Hastings C., Zajkowska Z., Mariani N., Nikkheslat N. (2021). Augmentation therapy with minocycline in treatment-resistant depression patients with low-grade peripheral inflammation: results from a double-blind randomised clinical trial. Neuropsychopharmacology.

[bib76] O'Connell N., Nicholson T., Wessely S., David A. (2020). Characteristics of patients with motor functional neurological disorder in a large UK mental health service: a case–control study. Psychol. Med..

[bib77] Ouzzani M., Hammady H., Fedorowicz Z., Elmagarmid A. (2016). Rayyan—a web and mobile app for systematic reviews. Syst. Rev..

[bib78] Peters M.D., Godfrey C.M., Khalil H., McInerney P., Parker D., Soares C.B. (2015). Guidance for conducting systematic scoping reviews. JBI Evidence Implementation.

[bib79] Peters M.D., Marnie C., Tricco A.C., Pollock D., Munn Z., Alexander L. (2020). Updated methodological guidance for the conduct of scoping reviews. JBI Evid. Synthesis.

[bib80] Raison C.L., Rutherford R.E., Woolwine B.J., Shuo C., Schettler P., Drake D.F. (2013). A randomized controlled trial of the tumor necrosis factor antagonist infliximab for treatment-resistant depression: the role of baseline inflammatory biomarkers. JAMA Psychiatr..

[bib81] Rapaport M.H., Delrahim K.K., Bresee C.J., Maddux R.E., Ahmadpour O., Dolnak D. (2005). Celecoxib augmentation of continuously ill patients with schizophrenia. Biol. Psychiatr..

[bib82] Redlich C., Berk M., Williams L.J., Sundquist J., Sundquist K., Li X. (2014). Statin use and risk of depression: a Swedish national cohort study. BMC Psychiatr..

[bib83] Roerink M.E., Bredie S.J.H., Heijnen M., Dinarello C.A., Knoop H., Van der Meer J.W.M. (2017). Cytokine inhibition in patients with chronic fatigue syndrome: a randomized trial. Ann. Intern. Med..

[bib84] Savitz J.B., Teague T.K., Misaki M., Macaluso M., Wurfel B.E., Meyer M. (2018). Treatment of bipolar depression with minocycline and/or aspirin: an adaptive, 2x2 double-blind, randomized, placebo-controlled, phase IIA clinical trial. Transl. Psychiatry.

[bib85] Sepanjnia K., Modabbernia A., Ashrafi M., Modabbernia M.J., Akhondzadeh S. (2012). Pioglitazone adjunctive therapy for moderate-to-severe major depressive disorder: randomized double-blind placebo-controlled trial. Neuropsychopharmacology.

[bib86] Sommer I.E., Gangadin S.S., De Witte L.D., Koops S., Van Baal C., Bahn S., Drexhage H., Van Haren N.E., Veling W., Bruggeman R., Martens P. (2021 Jul). Simvastatin augmentation for patients with early-phase schizophrenia-Spectrum disorders: a double-blind, randomized placebo-controlled trial. Schizophr. Bull..

[bib87] Strawbridge R., Arnone D., Danese A., Papadopoulos A., Vives A.H., Cleare A. (2015). Inflammation and clinical response to treatment in depression: a meta-analysis. Eur. Neuropsychopharmacol.

[bib88] Sugino H., Futamura T., Mitsumoto Y., Maeda K., Marunaka Y. (2009). Atypical antipsychotics suppress production of proinflammatory cytokines and up-regulate interleukin-10 in lipopolysaccharide-treated mice. Prog. Neuro Psychopharmacol. Biol. Psychiatr..

[bib89] Vane J.R., Botting R.M. (1998 Mar 30). Mechanism of action of nonsteroidal anti-inflammatory drugs. Am. J. Med..

[bib90] Vincenzi B., Stock S., Borba C.P., Cleary S.M., Oppenheim C.E., Petruzzi L.J. (2014). A randomized placebo-controlled pilot study of pravastatin as an adjunctive therapy in schizophrenia patients: effect on inflammation, psychopathology, cognition and lipid metabolism. Schizophr. Res..

[bib91] Weckmann A.L., Alcocer-Varela J. (1996 Oct 1). Cytokine inhibitors in autoimmune disease. InSeminars arthritis rheumatism.

[bib92] Weinberger J.F., Raison C.L., Rye D.B., Montague A.R., Woolwine B.J., Felger J.C. (2015). Inhibition of tumor necrosis factor improves sleep continuity in patients with treatment resistant depression and high inflammation. Brain Behav. Immun..

[bib93] Weiser M., Levi L., Burshtein S., Chirita R., Cirjaliu D., Gonen I. (2019). The effect of minocycline on symptoms in schizophrenia: results from a randomized controlled trial. Schizophr. Res..

[bib94] Weiser M., Zamora D., Levi L., Nastas I., Gonen I., Radu P., Matei V., Nacu A., Boronin L., Davidson M., Davis J.M. (2021 Jul). Adjunctive aspirin vs placebo in patients with schizophrenia: results of two randomized controlled trials. Schizophr. Bull..

[bib95] Woelfer M., Kasties V., Kahlfuss S., Walter M. (2019 Apr 1). The role of depressive subtypes within the neuroinflammation hypothesis of major depressive disorder. Neuroscience.

[bib96] Yudkin J.S., Stehouwer C., Emeis J., Coppack S. (1999). C-reactive protein in healthy subjects: associations with obesity, insulin resistance, and endothelial dysfunction: a potential role for cytokines originating from adipose tissue?. Arterioscler. Thromb. Vasc. Biol..

[bib97] Zdanowicz N.R.C., Jacques D., Lepiece B., Dubois T. (2017). Selective serotonergic (SSRI) versus noradrenergic (SNRI) reuptake inhibitors with and without acetylsalicylic acid in major depressive disorder. Psychiatr. Danub..

[bib98] Zeinoddini A., Sorayani M., Hassanzadeh E., Arbabi M., Farokhnia M., Salimi S. (2015). Pioglitazone adjunctive therapy for depressive episode of bipolar disorder: a randomized, double‐blind, placebo‐controlled trial. Depress. Anxiety.

[bib99] Zhang L., Zheng H., Wu R., Zhu F., Kosten T.R., Zhang X.Y. (2018). Minocycline adjunctive treatment to risperidone for negative symptoms in schizophrenia: association with pro-inflammatory cytokine levels. Prog. Neuro-Psychopharmacol. Biol. Psychiatry.

[bib100] Zhang Y., Shi H., Yang G., Yang Y., Li W., Song M., Shao M., Su X., Lv L. (2021 Nov 20). Associations between expression of indoleamine 2, 3-dioxygenase enzyme and inflammatory cytokines in patients with first-episode drug-naive Schizophrenia. Transl. Psychiatry.

