# Supplementary material for: Anti-inflammatory medications for the treatment of mental disorders: A scoping review
Source: Brain Behav Immun Health. 2022 Sep 19;26:100518. doi: 10.1016/j.bbih.2022.100518 (PMC9547233; doi:10.1016/j.bbih.2022.100518)
Supplement: Multimedia component 1 [file mmc1.docx]

**Appendix 1: Full Medline search strategy**

|  | **Antibiotics** |  | **NSAID** |
| --- | --- | --- | --- |
| 1 | antibiotic*.mp. | 151 | NSAID*.mp. |
| 2 | Amikacin.mp. | 152 | [ibuprofen.mp](http://ibuprofen.mp/). |
| 3 | Gentamicin.mp. | 153 | [naproxen.mp](http://naproxen.mp/). |
| 4 | Kanamycin.mp. | 154 | [ketoprofen.mp](http://ketoprofen.mp/). |
| 5 | Neomycin.mp. | 155 | [tolmetin.mp](http://tolmetin.mp/). |
| 6 | Netilmicin.mp. | 156 | [etodolac.mp](http://etodolac.mp/). |
| 7 | Tobramycin.mp. | 157 | [fenoprofen.mp](http://fenoprofen.mp/). |
| 8 | Paromomycin.mp. | 158 | [diclofenac.mp](http://diclofenac.mp/). |
| 9 | Streptomycin.mp. | 159 | [flurbiprofen.mp](http://flurbiprofen.mp/). |
| 10 | Spectinomycin.mp. | 160 | [misoprostol.mp](http://misoprostol.mp/). |
| 11 | Geldanamycin.mp. | 160 | [piroxicam.mp](http://piroxicam.mp/). |
| 12 | Herbimycin.mp. | 162 | [etodolac.mp](http://etodolac.mp/). |
| 13 | Rifaximin.mp. | 163 | [indomethacin.mp](http://indomethacin.mp/). |
| 14 | Loracarbef.mp. | 164 | [sulindac.mp](http://sulindac.mp/). |
| 15 | Ertapenem.mp. | 165 | [ketorolac.mp](http://ketorolac.mp/). |
| 16 | Doripenem.mp. | 166 | [meloxicam.mp](http://meloxicam.mp/). |
| 17 | Imipenem.mp. | 167 | [nabumetone.mp](http://nabumetone.mp/). |
| 18 | Cilastatin.mp. | 168 | [famotidine.mp](http://famotidine.mp/). |
| 19 | Meropenem.mp. | 169 | [esomeprazole.mp](http://esomeprazole.mp/). |
| 20 | Cefadroxil.mp. | 170 | [oxaprozin.mp](http://oxaprozin.mp/). |
| 21 | Cefazolin.mp. | 171 | mefenamic [acid.mp](http://acid.mp/). |
| 22 | Cephradine.mp. | 172 | [diflunisal.mp](http://diflunisal.mp/). |
| 23 | Cephapirin.mp. | 173 | [lansoprazole.mp](http://lansoprazole.mp/). |
| 24 | Cephalothin.mp. | 174 | Combine 151-173 with OR |
| 25 | Cefalexin.mp. |  | **Cytokine inhibitors** |
| 26 | Cefaclor.mp. | 175 | Cytokine inhibit*.mp. |
| 27 | Cefoxitin.mp. | 176 | [anti-cytokine.mp](http://anti-cytokine.mp/). |
| 28 | Cefotetan.mp. | 177 | [rilonacept.mp](http://rilonacept.mp/). |
| 29 | Cefamandole.mp. | 178 | [canakinumab.mp](http://canakinumab.mp/). |
| 30 | Cefmetazole.mp. | 179 | [reslizumab.mp](http://reslizumab.mp/). |
| 31 | Cefonicid.mp. | 180 | [brodalumab.mp](http://brodalumab.mp/). |
| 32 | Loracarbef.mp. | 181 | [ustekinumab.mp](http://ustekinumab.mp/). |
| 33 | Cefprozil.mp. | 182 | [anakinra.mp](http://anakinra.mp/). |
| 34 | Cefuroxime.mp. | 183 | [benralizumab.mp](http://benralizumab.mp/). |
| 35 | Cefixime.mp. | 184 | [guselkumab.mp](http://guselkumab.mp/). |
| 36 | Cefdinir.mp. | 185 | [tocilizumab.mp](http://tocilizumab.mp/). |
| 37 | Cefditoren.mp. | 186 | [dupilumab.mp](http://dupilumab.mp/). |
| 38 | Cefoperazone.mp. | 187 | [ixekizumab.mp](http://ixekizumab.mp/). |
| 39 | Cefotaxime.mp. | 188 | [mepolizumab.mp](http://mepolizumab.mp/). |
| 40 | Cefpodoxime.mp. | 189 | [secukinumab.mp](http://secukinumab.mp/). |
| 41 | Ceftazidime.mp. | 190 | [risankizumab.mp](http://risankizumab.mp/). |
| 42 | Ceftibuten.mp. | 191 | [tildrakizumab.mp](http://tildrakizumab.mp/). |
| 43 | Ceftizoxime.mp. | 192 | [sarilumab.mp](http://sarilumab.mp/). |
| 44 | Moxalactam.mp. | 193 | [satralizumab.mp](http://satralizumab.mp/). |
| 45 | Ceftriaxone.mp. | 194 | [basiliximab.mp](http://basiliximab.mp/). |
| 46 | Cefepime.mp. | 195 | [siltuximab.mp](http://siltuximab.mp/). |
| 47 | Ceftaroline [fosamil.mp](http://fosamil.mp/). | 196 | [daclizumab.mp](http://daclizumab.mp/). |
| 48 | Ceftobiprole.mp. | 197 | Combine 175-196 with OR |
| 49 | Teicoplanin.mp. |  | **Cox inhibitors** |
| 50 | Vancomycin.mp. | 198 | cox inhibit*.mp. |
| 51 | Telavancin.mp. | 199 | [amlodipine.mp](http://amlodipine.mp/). |
| 52 | Dalbavancin.mp. | 200 | [celecoxib.mp](http://celecoxib.mp/). |
| 53 | Oritavancin.mp. | 201 | [rofecoxib.mp](http://rofecoxib.mp/). |
| 54 | Clindamycin.mp. | 202 | [valdecoxib.mp](http://valdecoxib.mp/). |
| 55 | Lincomycin.mp. | 203 | Combine 198-202 with OR |
| 56 | Daptomycin.mp. |  | **TNF inhibitors** |
| 57 | Azithromycin.mp. | 204 | TNF inhibit* |
| 58 | Clarithromycin.mp. | 205 | Infliximab.mp. |
| 59 | Erythromycin.mp. | 206 | Adalimumab.mp. |
| 60 | Roxithromycin.mp. | 207 | Etanercept.mp. |
| 61 | Telithromycin.mp. | 208 | Certolizumab.mp. |
| 62 | Spiramycin.mp. | 209 | Golimumab.mp. |
| 63 | Fidaxomicin.mp. | 210 | Combine 204-209 with OR |
| 64 | Aztreonam.mp. |  | **Other medication terms** |
| 65 | Furazolidone.mp. | 211 | exp Anti-Inflammatory Agents, Non-Steroidal/ or exp Anti-Inflammatory Agents/ or Non-steroidal [anti-inflammatory.mp](http://anti-inflammatory.mp/). |
| 66 | Nitrofurantoin.mp. | 212 | Anti-inflammatory.mp. |
| 67 | Linezolid.mp. | 213 | corticosteroid*.mp. |
| 68 | Posizolid.mp. | 214 | [dexamethasone.mp](http://dexamethasone.mp/). |
| 69 | Radezolid.mp. | 215 | JAK inhibit*.mp. |
| 70 | Torezolid.mp. | 216 | Tofacitinib.mp. |
| 71 | Amoxicillin.mp. | 217 | baricitinib.mp. |
| 72 | Ampicillin.mp. | 218 | upadacitinib.mp. |
| 73 | Azlocillin.mp. | 219 | preficitinib.mp. |
| 74 | Dicloxacillin.mp. | 220 | filgotinib.mp. |
| 75 | Flucloxacillin.mp. | 221 | ruloxitinib .mp. |
| 76 | Mezlocillin.mp. | 222 | Fedratinib.mp. |
| 77 | Methicillin.mp. | 223 | VEGF-a.mp. or exp Vascular Endothelial Growth Factor A/ |
| 78 | Nafcillin.mp. | 224 | Combine 211-224 with OR |
| 79 | Oxacillin.mp. |  | **Mental health / MNYES** |
| 80 | Penicillin G.mp. | 225 | mental [health.mp](http://health.mp/). |
| 81 | Penicillin V.mp. | 226 | mental disorder*.mp. |
| 82 | Piperacillin.mp. | 227 | somatic symptom disorder*.mp. |
| 83 | Temocillin.mp. | 228 | conversion [disorder.mp](http://disorder.mp/). |
| 84 | Ticarcillin.mp. | 229 | functional neurological [disorder.mp](http://disorder.mp/). |
| 85 | Augmentin.mp. | 230 | functional movement disorder*.mp. |
| 86 | Unasyn.mp. | 231 | somatoform [disorder.mp](http://disorder.mp/). |
| 87 | Zosyn.mp. | 232 | [depression.mp](http://depression.mp/). |
| 88 | Timentin.mp. | 233 | PTSD.mp. |
| 89 | Bacitracin.mp. | 234 | Post-traumatic stress*.mp. |
| 90 | Colistin.mp. | 235 | Post traumatic stress*.mp. |
| 91 | Polymyxin B.mp. | 236 | depress*.mp. |
| 92 | Ciprofloxacin.mp. | 237 | anxiety*.mp. |
| 93 | Enoxacin.mp. | 238 | schizophreni*.mp. |
| 94 | Gatifloxacin.mp. | 239 | [psychosis.mp](http://psychosis.mp/). |
| 95 | Gemifloxacin.mp. | 240 | chronic fatigue [syndrome.mp](http://syndrome.mp/). |
| 96 | Levofloxacin.mp. | 241 | CFS.mp. |
| 97 | Lomefloxacin.mp. | 242 | [fatigue.mp](http://fatigue.mp/). |
| 98 | Moxifloxacin.mp. | 243 | chronic [pain.mp](http://pain.mp/). |
| 99 | Nadifloxacin.mp. | 244 | pain [disorder.mp](http://disorder.mp/). |
| 100 | Nalidixic [acid.mp](http://acid.mp/). | 245 | [fibromyalgia.mp](http://fibromyalgia.mp/). |
| 101 | Norfloxacin.mp. | 246 | persistent physical [symptoms.mp](http://symptoms.mp/). |
| 102 | Ofloxacin.mp. | 247 | functional [disorders.mp](http://disorders.mp/). |
| 103 | Trovafloxacin.mp. | 248 | bodily distress [disorder.mp](http://disorder.mp/). |
| 104 | Grepafloxacin.mp. | 249 | bodily distress [syndrome.mp](http://syndrome.mp/). |
| 105 | Sparfloxacin.mp. | 250 | irritable bowel [syndrome.mp](http://syndrome.mp/). |
| 106 | Temafloxacin.mp. | 251 | IBS.mp. |
| 107 | Mafenide.mp. | 252 | nonepileptic [seizures.mp](http://seizures.mp/). |
| 108 | Sulfacetamide.mp. | 253 | non-epileptic [seizures.mp](http://seizures.mp/). |
| 109 | Sulfadiazine.mp. | 254 | non epileptic attack [disorder.mp](http://disorder.mp/). |
| 110 | Silver [sulfadiazine.mp](http://sulfadiazine.mp/). | 255 | NEAD.mp. |
| 111 | Sulfadimethoxine.mp. | 256 | Combine 225-256 with OR |
| 112 | Sulfamethizole.mp. |  | **Additional keyword** |
| 113 | Sulfamethoxazole.mp. | 257 | treat*.mp. |
| 114 | Sulfanilimide.mp. |  | **Study types** |
| 115 | Sulfasalazine.mp. | 258 | (clinical adj trial).mp. |
| 116 | Sulfisoxazole.mp. | 259 | (Randomised adj trial).mp. |
| 117 | Trimethoprim-Sulfamethoxazole.mp. | 260 | (Randomised adj controlled).mp. |
| 118 | Co-trimoxazole.mp. | 261 | (Random* adj control*).mp. |
| 119 | Prontosil.mp. | 262 | Combine 258-262 with OR |
| 120 | Demeclocycline.mp. |  | **Combining groups** |
| 121 | Doxycycline.mp. | 263 | 150 OR 174 OR 197 OR 203 OR 210 OR 224 |
| 122 | Metacycline.mp. | 264 | 263 AND 256 AND 257 AND 262 |
| 123 | Minocycline.mp. |  | **Exclusions** |
| 124 | Oxytetracycline.mp. | 265 | 264 not [covid.mp](http://covid.mp/). |
| 125 | Tetracycline.mp. | 266 | 265 not [surgery.mp](http://surgery.mp/). |
| 126 | Clofazimine.mp. | 267 | 266 not [postoperative.mp](http://postoperative.mp/). |
| 127 | Dapsone.mp. | 268 | 267 not [arthritis.mp](http://arthritis.mp/). |
| 128 | Capreomycin.mp. | 269 | 268 not [orthodontic.mp](http://orthodontic.mp/). |
| 129 | Cycloserine.mp. | 270 | 269 not [tooth.mp](http://tooth.mp/). |
| 130 | Ethambutol.mp. | 271 | 270 not [tooth.mp](http://tooth.mp/). |
| 131 | Ethionamide.mp. |  | **Limit search** |
| 132 | Isoniazid.mp. | 272 | 271 limit to humans |
| 133 | Pyrazinamide.mp. |  |  |
| 134 | Rifampicin.mp. |  |  |
| 135 | Rifabutin.mp. |  |  |
| 136 | Rifapentine.mp. |  |  |
| 137 | Streptomycin.mp. |  |  |
| 138 | Arsphenamine.mp. |  |  |
| 139 | Chloramphenicol.mp. |  |  |
| 140 | Fosfomycin.mp. |  |  |
| 141 | Fusidic [acid.mp](http://acid.mp/). |  |  |
| 142 | Metronidazole.mp. |  |  |
| 143 | Mupirocin.mp. |  |  |
| 144 | Platensimycin.mp. |  |  |
| 145 | Quinupristin-Dalfopristin.mp. |  |  |
| 146 | Thiamphenicol.mp. |  |  |
| 147 | Tigecycline.mp. |  |  |
| 148 | Tinidazole.mp. |  |  |
| 149 | Trimethoprim.mp. |  |  |
| 150 | Combine 1-150 with OR |  |  |

**Appendix 2: Summary of identified systematic reviews assessing** **anti-inflammatory medications in the treatment of Mental Disorders (grouped by Mental Disorder and listed alphabetical by medication)**

| **Reference** | **Sample size** | **Condition** | **Medication** | **Primary papers included** | **All studies met current review criteria** | **Type of analysis** | **Primary outcome** | **Main conclusions** |
| --- | --- | --- | --- | --- | --- | --- | --- | --- |
| Na et al 2014^(1)^ | N=150  (4 RCTs) | Major depressive disorder | Celecoxib | Abbasi et al 2012, Muller et al 2006, Akhondzadch et al 2009, Majd et al 2015 | Yes | Meta-analysis | Change in HAM-D score at follow-up from baseline. Duration range 6-8 weeks. | Compared to placebo, sig. greater improvement in HAMD scores in Celecoxib group (WMD= 3.26, 95%CI=1.81 to 4.71), remission rates (OR=6.58, 95% CI=2.55 to 17.00) and response rates (OR= 6.49, 95%CI= 2.89 to 14.55). |
| Bai et al 2020^(2)^ | N=1610  (30 RCTs) | Major depressive disorder | Celecoxib (4 studies), Omega fatty acids (21 studies), Minocycline (3 studies), Piogltazone (1 study), Modafanil (2 studies), NAC (1 study) | Muller et al 2006; Akhondzadeh 2009, Abbasi et al 2012, Majd et al 2015, Sepanjnia et al 2012, Gougol et al 2015, Husain et al 2017, Dean et al 2017, | No (alternative treatments with fatty acid supplements used in 21 articles) | Meta-analysis and narrative review | Change in depression score form baseline to endpoints. Duration range: 4-16 weeks. | Meta-analysis of 26 studies suggested that compared to placebo, anti-inflammatory agents had a statistically significant antidepressant effect. SMD −0.55 (95% CI −0.75 to −0.35, p<0.00001). Sub-analysis revealed that this finding remained significant in inflammatory agent monotherapy (N=8, SMD -0.30 (95%CI -0.58-0.02), p=0.03) and as adjunctive treatment (n=18, SMD -0.70 (95% CI -0.97 - -0.43), p<0.00001). NSAID SMD −0.76 (-1.14 to -0.39) p<0.0001, N=4. Minocycline −0.79 (-1.29 to -0.28) p=.002, N=3 |
| Allison et al 2019^(3)^ | N=282  (8 RCT in total. 5 RCTs used anti-inflammatory medications) | Major depressive disorder | Celecoxib (2 studies), Infliximab (2 studies), Losmapimod. | Abbasi et al 2012, Raison et al 2013. | No (medications associated with mental health disorder, alternative treatments) | Narrative review | Change in depressive symptoms, change in inflammatory mediators, relationship between changes in scores of depressions and changes in inflammatory mediators. Duration range: 6-8 weeks. | Little evidence to support the role of anti-inflammatory agents in depression. Among the 5 studies which utilized anti-inflammatory medications, 1 showed a sig. reduction in inflammatory markers (IL-6) at follow-up (Abbasi et al) in treatment (Celecoxib) and control group. 2 showed no change and 2 did not measure follow-up inflammatory markers.  Only 1 study (Abassi et al) showed sig. reduction in HAM-D scores in treatment group compared to placebo group. 4 studies showed no changes in depressive scores in placebo vs controls. Many studies failed to stratify for baseline serum inflammatory markers in their analysis. |
| Rosenblat et al 2018^(4)^ | N=158  (3 RCTs) | Major depressive disorder | Minocycline | Hussain et al 2017 | No (reports open label studies, ongoing clinical trials and a case report) | Meta-analysis | Change in HAMD, MADRS total score at follow-up from baseline. Duration range 6-12 weeks. | Compared to placebo, sig. improvements in depression scores in Minocycline group. SMD= -0.78 [95% confidence interval (CI)−0.24 to−1.33 (P = 0.005). |
| Bavaresco et al 2020^(5)^ | N=201  (N=105 in meta-analysis)  (4 RCTs) | Major depressive disorder (treatment resistant) | Infliximab | Weinberger et al 2014. Raison et al 2013. Bekhbat et al 2018. Mehta et al 2013. | Yes | Meta-analysis and narrative review | Change in depression score (HAM-3). Duration range: 12 weeks | No sig. differences in change in HAM-D scores between adjuvant Infliximab versus placebo WMD = 1.90 (95% CI: −1.80, 5.60). |
| DeGiorgi et al 2021^(6)^ | N=  (5 RCTs) | Major depressive disorder | Statins | Ghanizadeh et al 2013  Gougol et al 2015  Haghighi2014 | No (included MDD post CABG and adolescent patients) | Meta-analysis | Mean value on HDRS-17 or MADRS at 8 weeks | Statins had a moderate effect in in improving depressive scores compared to placebo (SMD=-0.48, 95%CI=-0.74to-0.22), which was statistically significant |
| Fond et al 2014^(7)^ | Total sample not stated  (42 RCTs) | Major depressive disorder, Schizophrenia, Bipolar disorder | Celecoxib (13 studies), Acetylsalicylic acid (Aspirin) (16 studies), anti-TNFa (4 studies), Minocycline (9 studies) | Muller et al 2004, Muller et al 2010, Raison et al 2013, Abbasi et al 2012, Rapaport et al 2005 Akhondzadeh et al 2007, Laan et al 2010, | Yes | Narrative review | CGI  HAMD  Brief psychiatric rating scale  PANNS  SANS  Global assessment of functioning scale | In the context of depression, 5 (of 6) studies found a beneficial role of using anti-inflammatory as adjuncts in treatment of depression. This included 3 (of 3) studies for Celecoxib, 1 (of 1) study for high dose Aspirin, 1 (of 1) study for Minocycline and 0 (of 1) study for TNF-a.  In the context of schizophrenia, 6 (of 7) studies found a beneficial role of using anti-inflammatory as adjuncts in treatment of schizophrenia (sig. reduction in total PANNS score). This included 4 (of 4) studies for Celecoxib, 1 (of 1) study for high dose Aspirin and 2 (of 2) study for Minocycline. |
| Faridhosseini et al 2014^(8)^ | N=184  (5 RCTs)  (N=160 in meta-analysis; 4 RCTs) | Major depression, Bipolar disorder | Celecoxib | Abbasi et al 2012, Muller et al 2006, Akhondzadch et al 2009, Nery et al 2008. Majd et al 2015. | Yes | Meta analysis and narrative review | Change in HAM-D score at follow-up from baseline. Duration range 6-8 weeks. | Metanalysis showed that compared to placebo, adjunctive Celecoxib group showed sig decrease in HAMD score at 4 weeks (pooled difference in means = 3.3, 95%CI[1.2–5.3],p= 0.002) and 6 weeks (pooled difference in means = 3.43, 95%CI [1.9–4.9],p<0.0001). compared to placebo, greater response rate in add-on Celecoxib group (pooled OR=6.6, 95%CI [2.5–17],p<0.0001) and remission rates (pooled OR=6.6, 95%CI [2.7–15.9],p<0.0001). |
| Husain et al 2017^(9)^ | N=847  (14 RCTs) | Major depressive disorder, Bipolar disorder | Celecoxib, Minocycline, NAC, Infliximab, Acetylsalicylic acid (Aspirin) | Muller et al 2006, Akhondzadeh et al 2009, Abbasi et al 2012, Majid et al 2015, Raison et al 2013, Emadi-kouchak et al 2016, Nery et al 2008, Arabzadeh et al 2015 | No (alternative treatments) | Meta-analysis and narrative review | HAMD, IDS, BDI, MADRS, YMRS.  Duration range 6-24 weeks | Current research is inconclusive. compared to placebo, anti-inflammatory group showed sig. improvement in depression scores (SMD −0.71, 6 RCT, n=214, 95% CI −1.24 to −0.17, p=0.009) and reduction in manic symptoms  (SMD −0.72 (3 RCT, n=96, 95%  CI −1.31 to −0.13, p=0.02). These findings did not remain sig. when the change in depressive symptoms from baseline to end score was used as the dependant variable SMD=-0.52 (5 RCTs, n=194, 95% CI -1.01 to 0.05, p=0.07). |
| Bavaresco et al 2019^(10)^ | N=121  (3 RCTs) | Bipolar disorder | Celecoxib | Arabzadeh et al., 2015 | No (minimum age <18 in 2 studies) | Meta-analysis and narrative review | Change in Young Mania rating scale at baseline, 1, 3 and 6 weeks. | Meta-analysis showed that compared to placebo, adjunctive Celecoxib group showed sig. reduction in YMRS scores. Weighted mean difference was 5.54 (95% CI = 3.26-7.82); p < 0.001. |
| Rosenblat et al 2016^(11)^ | N=312  (10 RCT) | Bipolar disorder | Pioglitazone (1 study) NAC (2 studies), NSAIDS (2 studies), Omega 3 (6 studies). | Nery et al 2008 | No (Alternative treatment included). | Meta-analysis and narrative review | Change in HDRS, MADRS, IDSC, CGI from Follow-up to baseline.  Duration range 6-24 weeks | Compared to placebo, sig. improvements in depressive symptoms with anti-inflammatory medication adjunct (WMD=-0.40 (95% CI 0.14 to 0.65, p=0.002). Separately, NSAIDs showed no difference to placebo (SMD=0.02 95% CI= -0.52 to 0.56). |
| Schmidt et al 2019^(12)^ | N=130  (2 RCTs) | Schizophrenia | Acetylsalicylic acid (Aspirin) | Laan et al 2010  Dean et al 2017. | Yes | Meta-analysis | Change in PANNs total score at follow-up from baseline. Duration range 8-24 weeks. | Current research is inconclusive and holds weak evidence. Compared to placebo, at 24 weeks, sig. better PANSS total endpoint score in Aspirin adjunct group (Mean difference -6.56, 95%CI -12.04 to -1.08). No difference between groups before 24 weeks. |
| Sommer et al 2014^(13)^ | Total sample not stated  26 RCT | Schizophrenia | Acetylsalicylic acid (Aspirin) (2 studies), Celecoxib (5 studies), Davunetide (1 study), EPA and DHA fatty acids (7 studies), Oestrogen (7 studies), Minocycline (3 studies), Nacetylcysteine (1 study) | Muller et al 2002, Muller et al 2004, Rapaport et al 2005, Akhondzadeh et al 2007, Muller et al 2010. Laan et al 2010, Weiser et al 2012, Levkovitz et al 2010, Weiser et al 2012, | No (some studies include alternative treatment). | Meta -analysis and narrative review | Change in PANNS total score and brief psychiatric rating scale.  Duration range 5-12 weeks. | Compared to placebo, sig. improvement in PANNS total score amongst group Aspirin SMD 0.3 95% CI: 0.06–0.537, oestrogen SMD 0.51 (95% CI: 0.043–0.972), and NAC, SMD 0.45 (95% CI: 0.112–0.779).  Compared to placebo, no sig. improvement in PANNS total score for Celecoxib group SMD 0.15 (CI-0.669 - 0.959), Davunetide SMD −0.23 (95% CI: −0.65 to 0.19), DHA/EPA fatty SMD 0.09 (95% CI: −0.16 to 0.35) or Minocycline SMD 0.22 (95% CI: −0.39 to 0.82). |
| Zheng et al 2017^(14)^ | N=1252  (9 RCT) | Schizophrenia | Celecoxib | Muller et al 2002, Muller et al 2004, Muller et al 201, Rapaport et al 2005, Akhondzadeh et al 2007, | No (some studies included anti-inflammatory medication only as adjunct to other treatment) | Meta-analysis and narrative review | Change in PANNS total score and brief psychiatric rating scale.  Duration range 5-12 weeks | Compared to placebo, no sig. change in PANSS score in Celecoxib group among chronic patients. (SMD of -0.22, 95%CI -0.54 to 0.10 p=0.17). Sig. improvement observed only in patients with first episode psychosis (SMD=--0.47 (3 RCTs, n=180, 95%CI:-0.81 to -0.14 P<0.005). |
| Solmi et al 2017^(15)^ | N=413  (6 RCTs in meta-analysis) | Schizophrenia | Minocycline | Kelly et al 2015, Liu et al 2014, Chaudhry et al 2012, Levkovitz et al 2010 | No (included 1 open label study and 3 case series) | Meta-analysis and narrative review | PANNS total endpoint score  Duration range | Significantly better endpoint scores in Minocycline group compared to placebo in PANSS total score (SMD=–0.59; CI95%= –1.15, –0.03; p=0.04); PANSS negative score (SMD=–0.76; CI95%= –1.21, –0.31; p=0.001); SANS score (SMD=0. .60; CI95%= –0.94, –0.27; p<0.001). No sig difference in Endpoint PANSS positive symptom scores (p=0.13), depression rating scale scores (p=0.43), attention (p=0.47), memory (p=0.52). |
| Xiang et al 2017^(16)^ | N=472  (8 RCTs) | Schizophrenia | Minocycline | Chaudhry et al 2012, Kelly et al 2015, Levkovitz et al 2010, Zhang et al 2015, Zeng et al 2015, Kjodaeie-Ardakani et al 2014. | Yes | Meta-analysis | Change in PANNS total score and brief psychiatric rating scale.  Duration range 8-48 weeks | Compared to placebo, sig. improvement in total PANNS and BPRS in Minocycline group (SMD-0.64, (95%CI -1.02, -0.27), P=0.0008. Sig. improvement in PANNS negative and PANNS positive subscales. No significant difference in neurocognitive function |
| Nitta et al 2013^(17)^ | N=713  (8 RCTs) | Schizophrenia | NSAIDS: Celecoxib, Acetylsalicylic acid (Aspirin) | Muller et al 2002, Rapaport et al 2006, Akhondzadeh et al 2007, Muller et al 2010, Muller et al 2002, Muller et al 2006, Rappard et al 2004, Laan et al 201, Weiser et al 2012. | Yes | Meta-analysis | Change in PANNS total score at follow-up from baseline. Duration range 5-16 weeks. | Compared to placebo, no sig. improvement in total PANNS score in NSAID group WMD= 0.236 (95% CI: −0.484 to −0.012, P = .063). Secondary analysis revealed improvement in PANNS positive symptoms SMD −0.189 (95% CI: −0.373 to −0.005, P = .044), but not PANNS negative symptoms SMD 0.026 (95% CI: −0.169 to 0.117, P = .72). |
| Colour code: Green –strong evidence for anti-inflammatory agents; amber – inconclusive evidence or positive findings in subgroup only; red – no evidence to support anti-inflammatory agents | | | | | | | | |

**Appendix 3: Primary and secondary outcomes used by included articles**

| **Scale** | **Definition** |
| --- | --- |
| Young mania rating scale (YMRS) | The YMRS is an 11 item questionnaire designed to assess symptoms of mania over the last 48 hours. 7 Items are scaled on a score from 0 to 4, and 4 items are scaled from 0 to 8. Higher scores suggest more severe symptoms of mania. Scores can range from 0-60 and scores >20 are suggestive of mania.^(17, 18)^ |
| Hamilton depression rating scale -17 (HAMD17) | The HAMD is 17 item questionnaire designed to assess symptoms of depression over the last 7 days. Questions are scaled from 0-4. Higher scores are suggestive of more severe depressive symptoms. Scores can range from 0-52. Scores from 0-7 are considered normal, 8-13 mild; 14-18 moderate; 19-22 severe; and >22 severe depression.^(19, 20)^ |
| Montgomery-Asberg Depression Rating Scale (MADRS) | The MADRS is a 10 item questionnaire designed to assess symptoms of depression. Questions are scaled 0-6. Higher scores are suggestive of more severe symptoms of depression. Scores can range from 0-60. Scores from 0-6 are considered normal; 7-19 mild depression; 20-34 moderate depression; and >34 severe depression.^(21)^ |
| Positive and Negative Syndrome Scale (PANNS) | The PANNS is a 30 point questionnaire designed to assess the severity of symptoms in schizophrenia. There are 3 different domains: Positive scale, negative scale and general psychopathology scale. The positive and negative scale domains both consist of 7 items each. Each item is ranked from 1-7 based on severity. The general psychopathology scale consists of 19 items scaled from 1-7 assessing anxiety, depressive, somatic and cognitive symptoms. Higher scores are suggestive of more severe symptoms. The total score ranges from 30-210.^(22)^ |
| Clinical global impression scale (CGI) | The CGI is a 3 item questionnaire completed by clinicians, and is designed to assess symptom severity and response to treatment in individuals with mental health disorders currently enrolled in treatment studies. The 3 domains include symptom severity, clinical improvement scale and treatment efficacy index. Symptom severity is scaled from 1-7, higher scores are suggestive of more symptoms. Clinical improvement scale is ranked from 1-7. Lower scores are suggestive of greater improvement in symptoms. Efficacy index assesses both side-effects and therapeutic effect. Scores range from 0 (large improvement and no side-effects) to 16 (unchanged or worse and side-effects outweigh the therapeutic effects). There is no global score and each domain is interpreted separately.^(23)^ |
| Scale for the Assessment of Negative Symptoms (SANS) | The SANS is a 5 domain questionnaire designed to assess the severity of negative symptoms in schizophrenia. Each domain rates symptoms from 0-5 depending on severity. There are a total of 25 items. Scores range from 0-125. Higher scores are suggestive of a more severe burden of negative symptoms in schizophrenia.^(24, 25)^ |
| Brief Psychiatric Rating Scale (BPRS) | The BPRS is an 18 item questionnaire designed to globally measure psychiatric symptoms including depression, anxiety and psychotic symptoms. Each item is ranked on a scale from 1-7. Scores range from 18-126. Higher scores are suggestive of a mental health disorder and a higher burden and severity of psychiatric symptoms.^(26)^ |
| Checklist from individual strength (CIS) | The CIS is a 20 item questionnaire designed to assess behavioural, emotional, social, and cognitive symptoms of chronic fatigue syndrome. It consists of 4 domains: Subjective fatigue (8 items); Concentration (5 items); Motivation (4 items); and physical activity (3 items). Each item is rated on a scale from 1-7. Scores range from 20 to 140. Higher scores are suggestive of more severe symptoms.^(27)^ |

**References**

1. Na K-S, Lee KJ, Lee JS, Cho YS, Jung H-Y. Efficacy of adjunctive celecoxib treatment for patients with major depressive disorder: a meta-analysis. Progress in Neuro-psychopharmacology and Biological Psychiatry. 2014;48:79-85.
2. Bai S, Guo W, Feng Y, Deng H, Li G, Nie H, et al. Efficacy and safety of anti-inflammatory agents for the treatment of major depressive disorder: a systematic review and meta-analysis of randomised controlled trials. Journal of Neurology, Neurosurgery & Psychiatry. 2020;91(1):21-32.
3. Allison DJ, Sharma B, Timmons BW. The efficacy of anti-inflammatory treatment interventions on depression in individuals with major depressive disorder and high levels of inflammation: a systematic review of randomized clinical trials. Physiology & Behavior. 2019;207:104-12.
4. Rosenblat JD, McIntyre RS. Efficacy and tolerability of minocycline for depression: a systematic review and meta-analysis of clinical trials. Journal of Affective Disorders. 2018;227:219-25.
5. Bavaresco DV, Uggioni MLR, Ferraz SD, Marques RMM, Simon CS, Dagostin VS, et al. Efficacy of infliximab in treatment-resistant depression: A systematic review and meta-analysis. Pharmacology Biochemistry and Behavior. 2020;188:172838.
6. De Giorgi R, De Crescenzo F, Rizzo Pesci N, Martens M, Howard W, Cowen PJ, Harmer CJ. Statins for major depressive disorder: A systematic review and meta-analysis of randomized controlled trials. PloS one. 2021 Mar 30;16(3):e0249409.
7. Fond G, Hamdani N, Kapczinski F, Boukouaci W, Drancourt N, Dargel A, et al. Effectiveness and tolerance of anti‐inflammatory drugs' add‐on therapy in major mental disorders: a systematic qualitative review. Acta Psychiatrica Scandinavica. 2014;129(3):163-79.
8. Faridhosseini F, Sadeghi R, Farid L, Pourgholami M. Celecoxib: A new augmentation strategy for depressive mood episodes. A systematic review and meta‐analysis of randomized placebo‐controlled trials. Human Psychopharmacology: Clinical and Experimental. 2014;29(3):216-23.
9. Husain MI, Strawbridge R, Stokes PR, Young AH. Anti-inflammatory treatments for mood disorders: Systematic review and meta-analysis. Journal of Psychopharmacology. 2017;31(9):1137-48.
10. Bavaresco DV, Colonetti T, Grande AJ, Colom F, Valvassori SS, Quevedo J, et al. Efficacy of celecoxib adjunct treatment on bipolar disorder: systematic review and meta-analysis. CNS & Neurological Disorders-Drug Targets. 2019;18(1):19-28.
11. Rosenblat JD, Kakar R, Berk M, Kessing LV, Vinberg M, Baune BT, et al. Anti‐inflammatory agents in the treatment of bipolar depression: a systematic review and meta‐analysis. Bipolar Disorders. 2016;18(2):89-101.
12. Schmidt L, Phelps E, Friedel J, Shokraneh F. Acetylsalicylic acid (aspirin) for schizophrenia. Cochrane Database of Systematic Reviews. 2019(8).
13. Sommer IE, van Westrhenen R, Begemann MJ, de Witte LD, Leucht S, Kahn RS. Efficacy of anti-inflammatory agents to improve symptoms in patients with schizophrenia: an update. Schizophrenia Bulletin. 2014;40(1):181-91.
14. Zheng W, Cai D-B, Yang X-H, Ungvari GS, Ng CH, Mueller N, et al. Adjunctive celecoxib for schizophrenia: a meta-analysis of randomized, double-blind, placebo-controlled trials. Journal of Psychiatric Research. 2017;92:139-46.
15. Solmi M, Veronese N, Thapa N, Facchini S, Stubbs B, Fornaro M, et al. Systematic review and meta-analysis of the efficacy and safety of minocycline in schizophrenia. CNS Spectrums. 2017;22(5):415-26.
16. Xiang Y-Q, Zheng W, Wang S-B, Yang X-H, Cai D-B, Ng CH, et al. Adjunctive minocycline for schizophrenia: a meta-analysis of randomized controlled trials. European Neuropsychopharmacology. 2017;27(1):8-18.
17. Nitta M, Kishimoto T, Müller N, Weiser M, Davidson M, Kane JM, et al. Adjunctive use of nonsteroidal anti-inflammatory drugs for schizophrenia: a meta-analytic investigation of randomized controlled trials. Schizophrenia Bulletin. 2013;39(6):1230-41.
18. Young R, Biggs J, Ziegler V, Meyer D. Young mania rating scale. Handbook of Psychiatric Measures. 2000:540-2.
19. Young RC, Biggs JT, Ziegler VE, Meyer DA. A rating scale for mania: reliability, validity and sensitivity. The British Journal of Psychiatry. 1978;133(5):429-35.
20. Hamilton M. The Hamilton rating scale for depression. Assessment of depression: Springer; 1986. p. 143-52.
21. Williams JB. A structured interview guide for the Hamilton Depression Rating Scale. Archives of General Psychiatry. 1988;45(8):742-7.
22. Montgomery SA, Åsberg M. A new depression scale designed to be sensitive to change. The British Journal of Psychiatry. 1979;134(4):382-9.
23. Kay SR, Opler LA, Lindenmayer J-P. The positive and negative syndrome scale (PANSS): rationale and standardisation. The British Journal of Psychiatry. 1989;155(S7):59-65.
24. Guy W. ECDEU assessment manual for psychopharmacology: US Department of Health, Education, and Welfare, Public Health Service …; 1976.
25. Andreasen N. Scale for the Assessment of Negative Symptoms (SANS) Iowa City IA Univ. Iowa: 1984b[Google Scholar]. 1984.
26. Andreasen NC. The Scale for the Assessment of Negative Symptoms (SANS): conceptual and theoretical foundations. The British Journal of Psychiatry. 1989;155(S7):49-52.
27. Overall JE, Gorham DR. The brief psychiatric rating scale. Psychological Reports. 1962;10(3):799-812.
28. Vercoulen JH, Swanink CM, Fennis JF, Galama JM, van der Meer JW, Bleijenberg G. Dimensional assessment of chronic fatigue syndrome. Journal of Psychosomatic Research. 1994;38(5):383-92.
